# Molecular Crosstalk Between Intrauterine hCG and Endometrial Receptivity: Signalling Pathways, Immune Modulation, and Translational Perspectives in IVF

**DOI:** 10.3390/ijms27010278

**Published:** 2025-12-26

**Authors:** Charalampos Voros, Fotios Chatzinikolaou, Georgios Papadimas, Spyridon Polykalas, Aristotelis-Marios Koulakmanidis, Diamantis Athanasiou, Vasiliki Kanaka, Maria Kanaka, Kyriakos Bananis, Antonia Athanasiou, Aikaterini Athanasiou, Ioannis Papapanagiotou, Charalampos Tsimpoukelis, Maria Anastasia Daskalaki, Marianna Theodora, Nikolaos Thomakos, Panagiotis Antsaklis, Dimitrios Loutradis, Georgios Daskalakis

**Affiliations:** 1 1st Department of Obstetrics and Gynecology, ‘Alexandra’ General Hospital, National and Kapodistrian University of Athens, 80 Vasilissis Sofias Avenue, 11528 Athens, Greece; aristoteliskoulak@gmail.com (A.-M.K.); vasilikikanaka@outlook.com (V.K.); maira.kanaka24@gmail.com (M.K.); tsimpoukelischa@gmail.com (C.T.); md181341@students.euc.ac.cy (M.A.D.); martheodr@gmail.com (M.T.); thomakir@hotmail.com (N.T.); panosant@gmail.com (P.A.); 2 Laboratory of Forensic Medicine and Toxicology, School of Medicine, Aristotle University of Thessaloniki, 54124 Thessaloniki, Greece; fotischatzin@auth.gr (F.C.); loutradi@otenet.gr (D.L.); 3 Athens Medical School, National and Kapodistrian University of Athens, 15772 Athens, Greece; dr.georgepapadimas@gmail.com (G.P.); spyridonpolykalas@hotmail.com (S.P.); gpapamd@hotmail.com (I.P.); 4 IVF Athens Reproduction Center, 15123 Maroussi, Greece; diamathan16@gmail.com (D.A.); antoathan16@gmail.com (A.A.); diamathan17@gmail.com (A.A.); 5 King’s College Hospitals NHS Foundation Trust, London SE5 9RS, UK; kyriakos.bananis@nhs.net; 6 Fertility Institute-Assisted Reproduction Unit, Paster 15, 11528 Athens, Greece

**Keywords:** endometrial receptivity, embryo implantation, human chorionic gonadotropin, in vitro fertilisation, luteinising hormone/choriogonadotropin receptor, PI3K/Akt/eNOS signalling, stromal decidualisation

## Abstract

A limited period of endometrial receptivity is defined by molecular interactions between the embryo and maternal tissues, which are crucial for successful implantation. The results of clinical studies assessing intrauterine human chorionic gonadotropin (hCG) as an endometrial priming agent in in vitro fertilisation (IVF) have been inconsistent, markedly affected by dose, timing, and cycle context. This narrative review summarises molecular data demonstrating that hCG modulates immunological, stromal, endothelial, and epithelial compartments in a coordinated manner, affecting essential endometrial processes. hCG promotes adhesion competence and proliferation in the epithelium via a microRNA-regulated signalling axis (miR-126-3p–PIK3R2–PI3K/Akt). Intrauterine hCG promotes controlled apposition and invasion at the vascular interface by selectively strengthening endothelial junctional cohesion via VE-cadherin and CD146, without promoting angiogenesis. hCG collaborates with ERK/mTOR signalling to regulate autophagy and apoptosis, alters steroid–receptor networks in the stroma, initiates early decidual and survival markers (ACTA2, NOTCH1, complement C3), and enhances stress resistance. hCG modifies the immunological milieu by enhancing the activity of regulatory T cells and altering the distribution of uterine natural killer cells. This facilitates immunological tolerance and the remodelling of spiral arteries. These pleiotropic effects together enhance biomarkers and provide a scientific justification for context-dependent clinical responses, including patient-chosen, directed methods for the delivery of intrauterine hCG during IVF.

## 1. Introduction

The intricate process of embryo implantation requires exact temporal coordination between a receptive endometrium and a developmentally competent blastocyst, facilitated by ongoing bidirectional signalling at the fetal–maternal interface [1]. hCG, an early embryonic signal generated after implantation, is believed to function locally as a paracrine regulator of endometrial receptivity [2,3]. This complements its conventional function in luteal support. Functional luteinising hormone/hCG receptors (LHCGRs) are present in the endometrial epithelial, stromal, and endothelial compartments at the tissue level [4]. This allows hCG to affect the immunological characteristics of the decidual milieu, routes of stromal decidualisation (FOXO1, PRL, IGFBP1), endothelial cohesion, and vascular remodelling, as well as epithelial adhesion capabilities (pinopodes, integrin αvβ3) [5]. Intrauterine hCG perfusion before embryo transfer has been investigated as an endometrial “priming” method in assisted reproduction, resulting in variable clinical results. Certain meta-analyses suggest benefits under certain dose–timing settings, often 500 IU given just before transfer, whereas others, particularly in fresh transfer scenarios, do not demonstrate improvements in key outcomes [6,7]. This disparity underscores the need to study how hCG influences the biology of the endometrium to account for the variability in trial outcomes and to identify the subgroups most likely to derive benefit.

From a molecular perspective, hCG alters endometrial receptivity via coordinated epithelial stromal endothelial immune interactions, as shown by converging preclinical and translational studies [8]. The targeted suppression of miR-126-3p alleviates these effects, hence supporting a causal role of the established epithelial PI3K-Akt-eNOS axis (miR-126-3p-regulated) [9]. In vivo and cellular studies demonstrate that hCG increases miR-126-3p expression, suppresses PIK3R2, and activates the PI3K/Akt/eNOS pathway, thereby facilitating epithelial proliferation and improving barrier and adhesion preparedness [8]. Moreover, intrauterine hCG specifically elevates the endothelial cell–cell adhesion molecules VE-cadherin (CD144) and CD146 while maintaining the levels of VEGFR1/2 and general lineage markers constant. This suggests that endothelium stabilisation and vascular integrity may serve as a feasible strategy for improved apposition and controlled trophoblast invasion [10]. A paracrine signalling mechanism that synchronises epithelial receptivity with stromal remodelling aligns with hCG’s modification of a 48-analyte secretome in three-dimensional primary cultures, promoting macropinocytosis signals in the epithelium (HGF, M-CSF), inflammatory mediators in the stroma (CCL4, FGF2, IL-1β, IL-6, IL-17, VEGF), and epithelial–mesenchymal transition-like traits in mixed cultures [11]. Randomised donor results reveal that a single 500 IU bolus delivered at the day 3 equivalent timepoint promotes gland–stroma resynchronisation, increases ESR1/PGR levels, and upregulates early decidual/survival markers (α-SMA/ACTA2, C3, NOTCH1). This corresponds with primate data and indicates that stromal survival and decidual initiation are first targets of hCG [12]. The reinstatement of stressed decidual phenotypes is mediated by hCG via LHCGRs, resulting in the upregulation of HOXA10, ITGB3, LIF, FOXO1, and L-selectin ligands while also influencing the autophagy–apoptosis balance (↑Beclin1/LC3, ↓p62; Bax/Bcl-2 transitions) and activating ERK1/2–mTOR in stromal cells from patients with recurrent implantation failure (RIF). The inhibition of LHCGRs mitigates these effects, indicating that receptors are deficient in RIF [8].

The immunological re-education facilitated by hCG is equally significant. hCG influences uterine NK (uNK) cells, dendritic cells, macrophages, and regulatory T cells, facilitating spiral artery remodelling, trophoblast migration, and regulated inflammation. This creates a tolerogenic, pro-implantation milieu [1,3]. In RIF frozen–thawed cycles, pre-transfer hCG (usually 500 IU) is associated with enhanced implantation, clinical pregnancy, and live birth rates, as well as an increase in peripheral Tregs, especially in younger patients undergoing blastocyst transfer, thereby linking immune tolerance markers with clinical benefits [13]. Minor clinical investigations suggest that intrauterine hCG may modify the endometrial distribution of uNK cells in fertile donors [14]. The molecular layers provide a biological justification for the dose- and timing-dependent signals seen in meta-analyses [6,7]. Their explanation may elucidate why fresh cycles fail since the particular effects of hCG during the window may be less efficacious or misaligned with the progression of the endometrium induced by ovarian stimulation, supraphysiologic steroids, and alterations in the vascular and immunological systems.

Recent preclinical studies position hCG as superior to cell–cell communication circuits and central endometrial signalling hubs regarding its mechanism. In human endometrial epithelial cells in a mouse model of implantation failure, hCG elevated miR-126-3p, diminished PIK3R2 (which encodes the PI3K p85β regulatory subunit), and activated the PI3K/Akt/eNOS signalling pathway, leading to increased epithelial proliferation and receptivity markers [8,15]. The suppression of miR-126-3p attenuated these effects, indicating a causal sequence of miR-126-3p→PIK3R2→PI3K/Akt→eNOS [8]. The capacity of this axis to enhance eNOS phosphorylation (e.g., at Ser1177) and nitric oxide bioavailability may facilitate pinopode maturation, integrin activation (αvβ3), and epithelial adhesiveness at the implantation interface [16,17]. This may augment cGMP–PKG signalling, reorganise the actin cytoskeleton, and stabilise apical junctional complexes [2,8]. Simultaneously, FOXO1 and HOXA10/11 pathways influence the differentiation of epithelial cells and the communication between stromal and epithelial cells. Akt may influence cellular entry into the cell cycle and protein synthesis by interacting with ERK and mTOR [2]. By synchronising membrane trafficking, junctional architecture, and transcriptional receptivity with the constrained timeframe necessary for blastocyst apposition and attachment, these signals collectively transform hCG from a purely luteotropic hormone into a local organiser of epithelial preparedness [18].

Biopsies obtained during the implantation window indicated that this intervention enhances the cohesiveness of endothelial junctions at the vascular interface by upregulating VE-cadherin (CD144) and CD146 (MCAM), while leaving VEGFR1/2, CD31, and general epithelial/stromal/immune markers unchanged [10]. This pattern signifies improved barrier function, meticulously managed permeability, and reinforced endothelial–pericyte connection, since it represents junctional maturation rather than new angiogenesis. Bienert asserts that stronger adherens junctions may prevent shear-induced junctional failure, reduce paracellular leakage, and provide a more laminar microflow that stabilises the blastocyst and regulates the release of soluble mediators [10]. This framework asserts that NO produced downstream of hCG-PI3K/Akt-eNOS signalling enhances the transport of nutrients and cytokines without causing oedema or abnormal leakage, thus supporting junctional reinforcement by facilitating vasodilation and microvascular compliance [8]. The final outcome is a biophysically stable decidual microvasculature that maintains tissue integrity while permitting trophoblast exploration and controlled invasion. This equilibrium is crucial for effective implantation [19].

Moreover, three-dimensional primary cultures reveal that hCG reorganises the endometrial secretome in a compartment-specific way. Mixed cultures have hallmarks of epithelial–mesenchymal transition via factors, such as FGF2, HGF, IL-1β, and TNF, while stromal cells amplify signal transmission and inflammatory mediators, like CCL4, FGF2, IL-1β, IL-6, IL-17, and VEGF [20]. Epithelial cells exhibit markers of macropinocytosis and epithelial plasticity, including HGF and M-CSF [20]. IL-1β, IL-6, and IL-17 together provide a permissive, non-sterile inflammatory environment that facilitates tissue remodelling without inducing significant harm [21]. M-CSF may enhance the propensity of macrophages to migrate to the implantation site and alter their morphology to facilitate implantation. HGF may temporarily enhance the pliability of epithelial cells to facilitate the adhesion of the blastocyst [20]. EMT-like signs in mixed cultures demonstrate coordinated epithelial/stromal remodelling that mitigates strict epithelial restrictions and improves matrix–integrin interactions, while simultaneous VEGF promotes vascular adaptation [22]. The findings, together with the synthesis of the mini-review, designate hCG as a paracrine mediator that regulates vascular homeostasis, stromal decidual activation, and epithelial adhesion efficacy. By temporally modulating secreted signals and changes in cellular states, hCG facilitates the initiation and maintenance of the receptive window for early trophoblast decidua communication [2,20].

In addition to traditional receptivity pathways (e.g., integrins, LIF, HOXA10/11), hCG seems to affect stromal survival mechanisms and initial decidualisation processes, regulating tissue integrity and timing at the implantation site [2,23]. In a randomised donor study, a single intrauterine bolus of 500 IU hCG administered at the day 3 equivalent timepoint resulted in the realignment of gland–stroma “dating,” an increase in ESR1/PGR, and the induction of early decidual/survival markers α-SMA (ACTA2), complement C3, and NOTCH1 that aligned with findings from non-human primates [12]. α-SMA denotes a perivascular/myofibroblast-like stromal phenotype that improves peri-arteriolar stability, pericellular contractility, and matrix tension crucial for spiral artery remodelling and controlled trophoblast invasion [24]. The role of NOTCH in intercellular communication and its context-dependent regulation of differentiation and survival indicates that the induction of NOTCH1 is consistent with stromal fate decisions and decidual maturation; its upregulation is associated with the maintenance of a pro-survival decidual niche that endures mechanical and oxidative stress during early invasion [12]. The importance of complement C3 expression in the decidua within a controlled inflammatory–repair pathway is gaining recognition. Local C3/C3a signalling may provide a “permissive” inflammatory milieu crucial for implantation by regulating leukocyte migration and trophoblast orientation, while preventing direct lethal effects [25]. Despite significant steroid exposure during stimulated cycles, the increase in ESR1/PGR indicates that hCG may alter the timing of steroid–receptor transcriptional networks [26]. These networks later interact with cAMP–PKA and decidual transcription factors (e.g., FOXO1) to promote PRL/IGFBP1 and cell-state changes typical of responsive stroma. These characteristics suggest that hCG functions as a stromal synchroniser, connecting stromal biomechanical preparedness and survival signalling to epithelial temporal signals [12].

This perspective is supported and clarified by convergent cellular findings in RIF. Hormone reception is closely linked to the transcriptional and adhesion modules that govern embryo–epithelium “rolling,” stable adhesion, and stromal differentiation [27]. In primary endometrial stromal cells obtained from women experiencing RIF, hCG stimulated the production of HOXA10, ITGB3, FOXO1, LIF, and L-selectin ligands via the luteinising hormone/choriogonadotropin receptor [8]. LIF augments receptivity and epithelial communication, HOXA10 activates β3-integrin promoter activity and stromal receptivity, and ITGB3 and L-selectin ligands on the luminal surface increase the transition from tethering to stable attachment in the selectin–integrin switch [28]. Notably, hCG influenced stress adaptation and proteostasis. In conjunction with the activation of ERK1/2 and mTOR, the Bax/Bcl-2 balance shifted towards regulated survival, p62 decreased (indicating enhanced autophagic flux), and the autophagy markers Beclin1 and LC3 increased [8,29]. Although mTOR typically inhibits autophagy, these findings indicate the existence of time- and context-dependent mechanisms (such as ERK-mediated Beclin1 phosphorylation or varying mTORC1/2 interactions) through which restorative autophagy and transient growth factor signalling collaborate to eliminate damaged organelles and proteins, thereby maintaining the decidual phenotype under implantation stress [30]. A receptor-level susceptibility in RIF, linked to reduced baseline LHCGRs and impaired autophagy markers identified in RIF endometrium, may explain why some patient groups may not experience therapeutic advantages from hCG perfusion. The suppression of LHCGRs alleviated all these effects [8,31]. These findings conceptually support an hCG/LHCGR→autophagy–survival→receptivity axis that integrates adhesion gene programs (HOXA10/ITGB3/LIF), steroid–receptor timing (ESR1/PGR), and stress resilience (Beclin1/LC3–p62, Bax/Bcl-2) to establish a decidual microenvironment that is simultaneously receptive, mechanically competent, and resistant to injury characteristics that are likely to be limiting factors in RIF and may be recoverable with precisely administered, window-specific intrauterine hCG [32].

The second pillar is immune tolerance at the decidual interface. hCG influences both innate and adaptive compartments, altering the functional state and composition of uNK cells, enhancing regulatory T cell (Treg) programs, and modifying DC and macrophage phenotypes that facilitate trophoblast guidance and vascular remodelling [1,3,33]. Mechanistically, hCG can mitigate tissue-damaging inflammation during the implantation window by enhancing tolerogenic DC signatures (e.g., increased PD-L1/IDO activity), fostering IL-10/TGF-β environments that promote FOXP3^+^ regulatory T cells (Tregs), and diminishing Th1/Th17 polarisation [3,33]. Concurrently, uNK cells, predominantly characterised as CD56^bright^CD16 and exhibiting low cytotoxicity, demonstrate hCG-associated modifications that promote pro-angiogenic and tissue-constructive functions [34]. These encompass enhancing the secretion of angiopoietins, matrix remodelers, and members of the VEGF family (MMP-2/9), as well as refining chemokine axes (e.g., CXCL12/CXCR4) that regulate trophoblast migration [1,8]. Changes in KIR/HLA-C-relevant signalling contexts caused by hCG may also change the uNK licensing thresholds, which would allow for controlled spiral artery transformation while keeping the decidual integrity [8,33]. To create a “permissive but restrained” inflammatory tone that is necessary for embryo acceptance, hCG affects complement cues (like C3/C3a), helps DC-SIGN^+^ subsets that encourage Treg growth, and makes macrophages more likely to be in M2-like, pro-healing states on the myeloid side [1,3]. Giuliani asserts that this intervention has been clinically shown to alter the endometrial distribution of uNK in fertile donors, aligning with rapid in situ immune re-patterning [14]. Immunologic tolerance signatures correlate with measurable outcome improvements in RIF frozen–thawed cycles, wherein pre-transfer hCG levels generally around 500 IU are associated with enhanced implantation rates, clinical pregnancies, live births, and elevated peripheral Treg counts [35]. The effects are most pronounced in younger patients undergoing blastocyst transfer [13]. When taken together, these molecular and cellular findings provide a biologically sound rationale for the dose-, timing-, and population-dependent clinical signals observed in trial syntheses and meta-analyses [6,7].

The converging data predominantly indicates the pleiotropic role of intrauterine hCG in enhancing endometrial receptivity. To enhance microvascular cohesion and epithelial preparedness, (i) PI3K/Akt/eNOS and associated ERK/mTOR pathways are coordinated, (ii) stromal decidualisation and survival/autophagy mechanisms are re-established, thereby stabilising the biomechanical and metabolic environment, and (iii) a tolerogenic, pro-implantation immune environment is created, which equilibrates the uNK, DC/macrophage, and Treg pathways [8,10,20]. The field can progress towards precise hCG perfusion application once these mechanistic layers are correlated with clinical heterogeneity, encompassing age, ovarian stimulation context, embryo developmental stage, and fresh versus frozen cycles [36]. This means making sure that the volume and concentration are the same so that they can be absorbed into the uterus, finding the right dose and timing (usually about 500 IU given minutes to hours before transfer), and focusing on patients who are most likely to benefit from certain molecular or immunologic endometrial phenotypes, like less LHCGR signalling, less autophagic flux, more fragile endothelial junctions, or uNK/Treg disequilibrium [7,36]. This framework also leads to the development of biomarker-guided protocols (like transcriptomic/secretomic panels for HOXA10/ITGB3/LIF, VE-cadherin/CD146 signatures, and Treg/uNK indices) and adaptive trial designs that match hCG delivery with the unique window of implantation. This is how a commonly used adjunct can be turned into a stratified, mechanism-anchored IVF intervention.

## 2. Methods

We created a narrative review elucidating the processes by which intrauterine hCG may alter endometrial receptivity and the biology of implantation. This work does not constitute a systematic review or meta-analysis. However, in accordance with best-practice guidelines for narrative reviews and qualitative syntheses, we explicitly outlined our information sources, eligibility criteria, data elements, and evaluation procedures to improve transparency and reproducibility. The main goals were to connect molecular pathways to clinical variability in in vitro fertilisation and to integrate preclinical and clinical data from epithelial, stromal, endothelial, and immunological compartments.

Electronic searches were conducted in the Cochrane Library, Web of Science Core Collection, Embase, and MEDLINE/PubMed from the inception of the databases until 29 September 2025. We further used Scopus and the reference lists of significant articles and current reviews to ensure comprehensive coverage of preclinical mechanistic research. The PubMed methodology included mechanistic terminology, contextual administration, and regulated language with free-text phrases like hCG and endometrium. The subsequent example string is “chorionic gonadotropin” OR hCG OR “human chorionic gonadotropin” AND (endometrium OR endometrial OR decidua OR implantation OR “endometrial receptivity”) AND (intrauterine OR perfusion OR infusion OR “endometrial priming” OR “embryo transfer”), succeeded by pathway terms (PI3K, Akt, endothelial nitric oxide synthase, HOXA10, integrin, VE-cadherin, CD146, autophagy, Beclin1, LC3, p62, ERK, mTOR, regulatory T cell, uterine natural killer, dendritic cell, and macrophage). The search phase imposed no restrictions on the timing or kind of research. Conference abstracts were excluded from the synthesis after an originality assessment, unless a full paper was available.

Research examining peri-implantation processes using human ex vivo tissue and primary cell cultures exposed to hCG, as well as human clinical data on intrauterine hCG prior to embryo transfer, was considered suitable. Relevant animal models were used while concentrating on implantation biology or endometrial biology. In clinical investigations, a placebo or the absence of intrauterine hCG was considered a suitable comparator, but in experimental research, a vehicle or the absence of hCG was used. Alongside the operational specifics of hCG administration, including dosage in international units, concentration in international units per microlitre, volume in microlitres, and timing concerning embryo transfer or sampling, the clinical outcomes of interest encompassed implantation rate, clinical pregnancy, ongoing pregnancy, live birth, miscarriage, and ectopic pregnancy. The research examined canonical receptivity markers, including HOXA10/HOXA11, integrin β3, and leukaemia inhibitory factor; features of epithelial adhesion and pinopode formation; intracellular signalling pathways, such as phosphoinositide 3-kinase-Akt-endothelial nitric oxide synthase, extracellular signal-regulated kinase, and mechanistic target of rapamycin; and endothelial junctional molecules, like VE-cadherin and CD146. The research also examined autophagy apoptosis indicators, including Beclin1, LC3, p62, and Bax/Bcl-2, immune parameters, such as regulatory T cells and the phenotype and distribution of uterine natural killer cells; dendritic and macrophage polarisation, cytokines, and chemokines; and complement components, like C3. Only complete passages in English were used. Investigations not involving non-human subjects that lacked direct relevance to the endometrial, opinion articles devoid of main data, and systemic hCG investigations were excluded unless they provided a definitive mechanistic insight into the endometrium.

Two reviewers separately evaluated the titles, abstracts, and complete texts according to the established criteria; any discrepancies were resolved via discussion. We used a reference manager to eliminate duplicates and verified them manually. In instances of numerous reports from the same cohort or experiment, the most comprehensive or current report was selected as the primary source. Previous analogous papers were only used to supplement missing mechanistic data. The data extraction used a standardised template that included the research design, model or setting, sample size, hCG dosage parameters, comparison, and outcomes. Mechanistic extraction documented the cellular compartment under investigation, the direction and magnitude of effects, and assay techniques, including quantitative polymerase chain reaction, Western blotting, immunohistochemistry, flow cytometry, enzyme-linked immunosorbent assay, and sequencing, as relevant. We noted the directionality of effects while recognising a restriction in cases when figures were quantitatively interpretable but lacked corresponding numerical data.

Critical evaluation tailored for these purposes was used to contextualise rather than exclude research, since this represents a narrative synthesis of diverse information. We used the SYRCLE tool and essential ARRIVE reporting items to evaluate animal studies, the major domains of ROBINS-I for non-randomised clinical studies, and the core domains of the revised Cochrane risk of bias tool for randomised trials to assess randomised trials. We assessed the methodological rigour of cellular and tissue investigations, including technical and biological replication, time-course design, antibody validation, normalisation procedures, concentration–response analysis, and statistical methods. Qualitative descriptions of the issues were used to assess the strength of the mechanistic conclusions.

Four interrelated biological themes, epithelial signalling and adhesion preparedness, endothelial junctional integrity and microvascular equilibrium, stromal decidualisation and survival with proteostasis regulation, and immune tolerance at the fetal–maternal interface, were subsequently utilised to synthesise the evidence through a theory-driven mapping. The concordance across models, the triangulated directionality of effects, and the associated pathway activation in clinical contexts, including fresh vs. frozen cycles, embryo developmental stage, ovarian stimulation environment, and patient age, were examined within each topic. To determine the circumstances in which intrauterine hCG is most beneficial and to suggest biomarker-guided, patient-selected applications for future prospective evaluation, we examined mechanistic signals in conjunction with clinical variability.

## 3. Endometrial Receptivity: A Molecular Overview

Endometrial receptivity is the brief period during which the luminal epithelium and the underlying stroma acquire the requisite biochemical and biophysical characteristics for the blastocyst to attach, adhere, and penetrate in a regulated manner [37]. This receptive state, characterised by alterations in blood vessels and the immune system that facilitate trophoblast guidance, also illustrates the interplay between steroid-driven transcriptional programs and paracrine signalling in influencing LIF, homeobox regulators, such as HOXA10/HOXA11, and adhesion mechanisms, including the αvβ3 integrin and pinopode maturation [2]. LIF interacts with the gp130 receptor complex to activate Janus kinase/signal transducer and activator of transcription-3 (JAK/STAT3) at the epithelial surface. This corresponds with progesterone receptor-dependent pathways and promotes cytoskeletal remodelling and adhesion ligand expression [38]. The renowned “adhesion switch,” characterised by the localised remodelling of anti-adhesive glycocalyx components and the bridging of osteopontin (SPP1) to αvβ3 integrin in the trophectoderm, is mediated by HOXA10, which is stimulated by progesterone through Indian hedgehog (IHH)–HAND2 signalling and transcriptionally activates β3-integrin along with a collection of receptivity genes [39]. Ezrin–radixin–moesin scaffolds, annexins, and aquaporin-3 facilitate the stabilisation of pinopodes, which are apical, actin-rich protrusions, regulated by progesterone/LIF. This establishes a high-affinity landing zone that facilitates L-selectin ligand-mediated tethering and robust integrin binding [40]. Prostaglandin E2 and cyclic adenosine monophosphate augment local second-messenger pathways that prime the epithelium for interaction with the blastocyst, whereas heparin-binding EGF-like growth factor (HB-EGF) and WNT/β-catenin signalling further modulate epithelial plasticity and membrane dynamics [2].

Progesterone and cyclic adenosine monophosphate promote decidualisation in the stroma, marked by the production of prolactin, insulin-like growth factor binding protein-1, and FOXO1 [41]. This process transforms fibroblasts into decidual cells that release chemicals, withstand stress, regulate matrix turnover, and provide metabolic buffering for the implanting conceptus [42]. The vascular endothelial growth factor-A/VEGFR2 and angiopoietin-Tie2 pathways regulate microvascular perfusion and permeability to satisfy the metabolic and signalling requirements of the peri-implantation environment [43]. Concurrently, these alterations in the stroma transmit signals to the epithelium via LIF and interleukin networks to maintain synchronised timing [2]. In human three-dimensional endometrial cultures subjected to embryonic signals, chemokines, like CXCL12 and CCL2, interact with cytokines to provide guidance cues for trophoblast and myeloid cell migration, thereby producing a permissive, non-destructive inflammatory environment [20]. The integrin–ligand structure (αvβ3–osteopontin), transcriptional activation (HOXA10/HOXA11), and STAT3-mediated signalling (LIF) work together to generate a temporary but highly coordinated receptive phenotype that enables accurate apposition, adhesion, and controlled invasion of the blastocyst [44].

The cytokine and chemokine dense milieu around the receptive phenotype precisely modulates stromal differentiation, extracellular matrix remodelling, and epithelium plasticity via intimately interconnected receptor–ligand interactions [45]. Three-dimensional primary cultures of human endometrium, consisting of epithelial, stromal, and mixed compartments, produce unique yet convergent secretomes enriched with chemokine CCL4, fibroblast growth factor-2, interleukins-1β, -6, and -17, tumour necrosis factor, vascular endothelial growth factor, hepatocyte growth factor, and macrophage colony-stimulating factor, each fulfilling a distinct signalling role [20]. To enable transitions from tethering to adhesion, parallel actin regulatory modules adjust the stiffness of the cortex. HGF–c-MET activates epithelial membrane ruffling and macropinocytosis via the Rac1/PI3K/Pak1 pathway, facilitating rapid remodelling of the apical surface and integrin recycling via Rab11-dependent endosomes [46]. CSF1 stimulates CSF1R on both resident and recruited macrophages to promote tissue-constructive phenotypes that release matrix metalloproteinases (MMP-2/-9) and tissue inhibitors of metalloproteinases in ratios favourable for controlled basement membrane loosening [47]. CCL4–CCR5 signalling influences leukocyte migration and the impact of inflammation on trophoblasts, while IL-1β initiates NF-κB-dependent transcription of adhesion and protease genes in the stroma and epithelium [48]. TNF enhances NF-κB/JNK signalling, accelerating the turnover of junctional components essential for receptive plasticity in low-grade stimuli. IL-6, via gp130/JAK–STAT3, promotes epithelial differentiation and survival, whereas IL-17, via ACT1–TRAF6, stimulates neutrophil-attracting and matrix remodelling pathways without triggering lethal inflammation. To synchronise microvascular perfusion with the metabolic and signalling requirements of the peri-implantation niche, VEGF engages with VEGFR2 to modulate endothelial fenestration and shear responsiveness [20].

These paracrine circuits interconnect with the transcriptional networks of progesterone and oestrogen receptors to establish a molecular foundation for a precisely time-regulated receptive state, essential for epithelial adhesion competence to align with stromal decidual initiation [2,20]. Cytokine-induced STAT3 and NF-κB pulses promote the timely expression of adhesion ligands (e.g., osteopontin interacting with αvβ3) and matrix regulators, whereas progesterone-primed HOX and FOXO modules establish a baseline of transcriptional permissiveness [49]. Lactate generated locally and hypoxia-inducible factor-1α regulate redox and angiogenic balance to stabilise the interface, while chemokine pathways, such as CXCL12–CXCR4 and CCL2–CCR2, provide directional signals for the migration of trophoblast and myeloid cells [50]. Complement fragments (C3a/C5a) and bioactive lipids (e.g., sphingosine-1-phosphate) influence leukocyte diapedesis and endothelial barrier function. Selective glycocalyx modification, including heparanase-mediated trimming, enhances L-selectin-mediated rolling prior to integrin activation. In this integrated system, hCG-induced secretomes function as a temporal regulator [51]. Stromal cytokines facilitate decidual gene expression and proteostasis, endothelial and angiogenic signals maintain microvascular equilibrium, and macropinocytosis and cytoskeletal dynamics preserve the epithelial surface for optimal high-affinity adhesion. These elements together provide a transient, meticulously orchestrated environment conducive to blastocyst apposition, adhesion, and regulated invasion [2,20].

Receptivity relies on both the integrity of endothelial junctions and the architecture of microvessels. For the blastocyst to attach and the trophoblast to probe the decidua without causing swelling, bleeding, or barrier failure, the peri-implantation endometrium needs to have mature adherens junctions, exactly calibrated permeability, and sprouting angiogenesis [52]. In vivo human studies indicate that intrauterine hCG administration during the implantation window specifically elevates VE-cadherin (CD144) and CD146 (MCAM) levels on the endometrial endothelium, without altering VEGFR1/2 or pan-endothelial CD31 levels [10]. VE-cadherin complexes attach to cortical actin using β-catenin and α-catenin. At the same time, p120-catenin stops VE-cadherin from being taken up by cells. Stabilising these complexes via hCG-conditioned signalling is anticipated to diminish paracellular leakage, enhance Rap1-mediated junctional tightening (cAMP–Epac–Rap1), and suppress Src–RhoA/ROCK–myosin light chain phosphorylation [53]. CD146, a junctional immunoglobulin superfamily receptor that is abundant at endothelial–endothelial and endothelial–pericyte contacts, collaborates with VE-cadherin to enhance lateral cohesion, regulate leukocyte diapedesis, and modify matrix adhesion. This adds another molecular brake to the formation of gaps caused by shear and the free entry of trophoblasts [10]. Angiopoietin-1/Tie2 and N-cadherin-mediated endothelial pericyte anchoring, along with sphingosine-1-phosphate/S1PR1 cues that promote barrier assembly, signals that work in concert with the cadherin axis to sustain a low-noise, receptive microcirculation, all contribute to perivascular stabilisation [54].

To preserve a receptive vascular bed, NO, generated subsequent to hCG-induced PI3K/Akt activation and endothelial nitric oxide synthase phosphorylation (e.g., at Ser1177), augments microflow and shear at the interface and bolsters junctional cohesion [8,10]. NO reduces actomyosin contractility via soluble guanylate cyclase–cGMP–protein kinase G by activating myosin light chain phosphatase. This increases the flexibility of capillaries while keeping junctions strong [55]. NO also lowers VCAM-1 and ICAM-1 levels, which stops endothelial activation, lowers reactive oxygen species levels by improving eNOS coupling, and stabilises tight-junction scaffolds (occludin/claudins/ZO-1), all of which help to reduce oedema [55]. Shear-responsive transcription factors, such as KLF2/KLF4, which are activated by NO-permissive flow, further promote an anti-inflammatory, antithrombotic endothelial phenotype. Meanwhile, controlled VEGF-A/VEGFR2 signalling (which has not changed in abundance in Bienert) is still set up for functional tuning rather than growth. The end result is vascular poise, which aligns the mechanics of the endothelium with the time-sensitive needs of implantation through effective nutrient and cytokine exchange, laminar microhemodynamics, and selective permeability that keep trophoblasts from going too deep into the tissue [8,10].

Immune processes constitute the third axis of receptivity, promoting positive remodelling and reinforcing tolerance. The decidual interface contains many uNK cells exhibiting modest cytotoxicity, regulatory Tregs, dendritic cells, and macrophages [56]. These cells collaborate to modify the spiral arteries, remodel the matrix, and establish chemokine gradients that direct trophoblast migration [1,3]. By secreting members of the vascular endothelial growth factor family, angiopoietins, matrix metalloproteinases, and guidance cues, uNK cells in this niche engage in a pro-angiogenic, tissue-constructive program that facilitates endothelial loosening and regulated vascular remodelling while reducing perforin/granzyme-mediated cytotoxicity [57]. KIR interactions with extravillous trophoblast human leukocyte antigen-C form a regulatory framework that influences cytokine production and vascular responses in a context-dependent and genotype-dependent manner, thereby augmenting their responsiveness [1,33]. Parallel Treg network FOXP3^+^ cells influenced by transforming growth factor-β and interleukin-10 mitigate excessive T-helper-1/T-helper-17 inflammation, stabilise stress responses in stromal and epithelial cells, and provide a local milieu conducive to embryo acceptance [3]. To reduce effector T cell activation, regulate tryptophan metabolism, and coordinate debris clearance and matrix renewal necessary for non-destructive invasion, dendritic cells and macrophages assume tolerogenic, M2-like phenotypes marked by increased interleukin-10, programmed death-ligand 1, and indoleamine-2,3-dioxygenase activity [3,33,58]. hCG is becoming acknowledged as a precursor signal that predisposes each of these compartments to conditions conducive to implantation. This is achieved by augmenting dendritic/macrophage pathways that emphasise vascular remodelling and trophoblast orientation over inflammation, elevating Tregs in interleukin-10/transforming growth factor-β contexts, and reprogramming uNK cells to exhibit pro-angiogenic, matrix-modulating traits with reduced cytotoxicity [1,3,33]. The tuning of these innate-adaptive circuits by hCG establishes a permissive but rigorously regulated inflammatory state that facilitates the development of the placental bed and sustained implantation. This milieu is suitable for remodelling and invasion. Nevertheless, it is also constrained to maintain barrier integrity and fetal–maternal tolerance [3,59]. Table 1 delineates the compartment-specific targets of intrauterine hCG, their immediate signalling effects, subsequent programs, quantifiable PD readouts, and practical consequences for implantation. This is accomplished to integrate the epithelial, endothelial, stromal, and immunological components into a practical format for therapeutic use.

Table 1 consolidates the multi-axis footprint of intrauterine hCG into a singular framework. By downregulating miR-126-3p→PIK3R2, hCG alters the equilibrium of PI3K/Akt→eNOS in the luminal epithelium, resulting in a rapid release of NO that relaxes the cortical actin network and accelerates the shift from apposition to adhesion. Epithelial PGF_2_α concurrently stimulates ADAM17-mediated shedding of EREG/HB-EGF, effectively priming the adjacent stroma. The development of junctions in the microvascular endothelium (characterised by the acquisition of VE-cadherin/CD146 without the overexpression of VEGFR1/2) facilitates steady shear exchange without oedema. This design facilitates regulated, non-invasive trophoblast ingress. The remodelled EV cargo transmits receptivity signals across compartments, whereas initial survival and decidual cues (ACTA2, NOTCH1, C3) correlate with regained proteostasis (Beclin1/LC3 with a drop in p62) and epigenetic priming (H3K27Ac at FOXO1/HOXA10/HAND2) inside the stroma. The expansion and functional inclination of FOXP3 Tregs and uNK phenotypes that promote vascular remodelling lead to a transition in the immunological interface towards tolerance. The specified PD readouts (e.g., epithelial p-Akt/p-eNOS or miR-126-3p/PIK3R2, endothelial VE-cadherin/CD146 continuity, stromal ACTA2/NOTCH1/C3 and LC3/p62 balance, immunological Treg/uNK metrics) provide the verification of on-target presence in uterine fluid, endometrial aspirates, or peripheral blood. The interaction of these layers explains why the timely application of planned FET, particularly with D5 blastocysts, consistently converts mechanistic engagement into clinical advantage and validates minutes-scale, small-volume/high-concentration dosing right before embryo transfer. Table 2 encapsulates preclinical and translational investigations of intrauterine hCG, including animal models, primary human tissues, and ex vivo systems. It also has details about their exposures, outcomes, directions of impact, and significant warnings. This provides a definitive dataset to support the mechanistic perspective.

Table 2 demonstrates a uniform signal applicable across all platforms. hCG stimulates stromal survival and decidual processes while reinstating autophagy proteostasis. It also strengthens endothelial adherens junctions without inducing angiogenic sprouting, reconfigures the immunological interface towards tolerance, and rapidly activates the epithelial PI3K/Akt→eNOS pathway and the epithelial PGF_2_α→ADAM17→EREG/HB-EGF cascade. Recent studies indicate that epigenetic enhancer activation (H3K27Ac) at essential decidual loci and extracellular vesicle cargo remodelling show that a transient hCG pulse may trigger prolonged paracrine and transcriptional preparedness. The integration of orthogonal models improves causal inference and clarifies why micro-volume, high-concentration dosage just before embryo transfer in planned frozen embryo transfer is the best biologically plausible approach to transform mechanistic engagement into clinical benefit. Nevertheless, limitations are common in translational research (restricted sample size, in vitro duration, surrogate outcomes).

## 4. Immune Crosstalk and hCG

For implantation to succeed, the immune system must be both permissive and restricted. It must possess the capacity to withstand the semiallogeneic embryo while also maintaining sufficient activity to facilitate matrix and vascular remodelling [63]. The decidual interface contains many uNK cells with limited cytotoxicity (CD56^bright^CD16^−^), regulatory Tregs, dendritic cells, and macrophages [33]. These cells collaborate to regulate the metamorphosis of spiral arteries, the migration of trophoblasts directed by chemokines, and the turnover of the matrix without inducing harm [1,3]. Decidual stromal cAMP–PKA–FOXO1 signalling initiates the synthesis of immunoregulatory mediators, including prostaglandin E2 and galectin-1, which suppress Th1/Th17 polarisation and mitigate tissue stress [64]. Conversely, progesterone and oestradiol molecularly imprint this niche via receptor-dependent transcription, leading to the overexpression of IL-15 (promoting uNK survival and homeostasis), CXCL12 (directing uNK and extravillous trophoblast localisation), and LIF [65]. While local complement activity persists, complement regulators (CD46, CD55, and CD59) are increased to prevent bystander lysis. Instead, they are altered to provide chemotactic and clearance signals that facilitate implantation [66]. Simultaneously, negative regulators, such as A20/TNFAIP3, inhibit the excessive activation of toll-like receptors, hence preventing sterile inflammation induced by remodelling. hCG serves as an upstream biasing signal within this immuno-endocrine framework, establishing a distinctive uterine immune profile that differentiates it from pathogen-defense mucosae and connects hormone-responsive transcription to immunological checkpoints [1,3].

uNK cells, characterised by a specific molecular specialisation, serve as the primary innate framework for vascular adaptation and trophoblast direction [34]. In addition to chemokines, such as CXCL8 and CXCL10, that regulate cellular movement, uNK cells secrete VEGF-A, VEGF-C, placenta growth factor, angiopoietins, and matrix metalloproteinases (MMP-2 and MMP-9). To protect the endothelium, perforin/granzyme pathways are epigenetically suppressed [1]. The interactions between KIR and extravillous trophoblast HLA-C establish their activation threshold, resulting in genotype-dependent licensing between the mother and foetus that alters GM-CSF, IFN-γ, and angiogenic responses [33,67]. hCG interacts with this circuitry both directly and indirectly. To maintain uNK viability and pro-angiogenic programming, hCG engages with LHCGR-expressing stromal and epithelial compartments, augmenting paracrine signals (IL-15, CXCL12, HB-EGF) [68]. Activation of endothelial PI3K/Akt–eNOS creates a nitric oxide-permissive, laminar shear environment that promotes the vascular-support identity of uNK cells instead of triggering cytotoxic activation [8,10]. An intrauterine hCG infusion during the embryo transfer window in viable donors elicits an alteration in uNK topology inside the endometrium, consistent with fast niche re-patterning [14]. Supplementary stromal and endothelial ligands (Jagged1/2–Notch) and metabolic signals (kynurenine–AHR from tolerogenic dendritic cells) further delineate uNK subsets (uNK1/2/3) into matrix-modulating, angiogenesis-capable phenotypes. This immune-vascular synergy facilitates controlled trophoblast infiltration and regulated spiral artery remodelling via constricted endothelial junctions (VE-cadherin/CD146), enhanced nitric oxide bioavailability, and uterine natural killer cell-mediated angiogenic reconfiguration [8,10,14].

FOXP3+ Tregs maintain beneficial inflammation essential for implantation while inhibiting effector responses, hence promoting adaptive tolerance. Decidual Tregs exhibiting checkpoint dominance and epigenetic stabilisation demonstrate elevated Helios expression, consistent FOXP3 TSDR demethylation, and increased levels of CTLA-4, TIGIT, and PD-1 [3]. Low-dose IL-2–STAT5 signalling and IL-10/TGF-β–SMAD2/3 signalling facilitate their proliferation and survival. They also modify APCs via CTLA-4–CD80/86 to induce indoleamine-2,3-dioxygenase, which depletes tryptophan and produces kynurenines that interact with the aryl-hydrocarbon receptor to maintain Treg lineage stability and prevent Th1/Th17 skewing. hCG augments this axis by facilitating IL-10/TGF-β settings and increasing tolerogenic dendritic cells and macrophages. It also interacts with ERK/mTOR pathways that govern Treg metabolic fitness (OXPHOS/FAO bias) and persistence [3,33]. Pre-transfer intrauterine hCG, often about 500 IU, is clinically correlated with enhanced implantation rates, clinical pregnancies, and live babies in instances of recurrent implantation failure during frozen–thawed cycles. It further enhances peripheral Tregs. The effects are especially pronounced in younger individuals who get blastocysts [13]. These findings indicate simultaneous adaptive recalibration. The proliferation of Tregs reduces rejection signals, especially at the trophoblast decidua interface, peaking when stromal decidual preparedness and epithelial adhesion capability are optimised [69]. The number and function of Tregs, IDO1 activity, and checkpoint activation (CTLA-4/PD-1) are acknowledged as possible indicators that connect hCG-induced immunological tolerance to outcomes [3,69].

In addition to uterine natural killer cells and regulatory T cells, hCG influences additional immunological subpopulations that facilitate implantation-associated tolerance. Experimental studies demonstrate that hCG facilitates the development and functional maturation of MDSCs, which exhibit considerable immunosuppressive effects by suppressing effector T cell proliferation, generating nitric oxide, and expressing arginase-1. MDSCs are recognised for their role in preventing the immune system from attacking the foetus during early pregnancy and accumulating at the maternal–fetal interface.

By inhibiting Th17-associated inflammatory responses and indirectly augmenting Treg stability, hCG modifies the balance between Th17 cells and regulatory T cells, fostering a tolerogenic shift. The balance of Th17 and Treg cells is crucial during implantation, since excessive Th17 activity has been associated with early pregnancy loss and implantation failure. Recent findings indicate that hCG influences immunological memory T cell compartments by reducing excessive immune activation upon embryo identification, hence lessening pro-inflammatory recall responses and fostering a tolerant memory phenotype. These activities position hCG at the forefront of many immune regulatory pathways, transcending traditional uNK–Treg interactions, therefore promoting a controlled, implantation-supportive immune milieu.

Myeloid cells serve as intermediaries in remodelling and tolerance, uniting tissue restoration with immune suppression [70]. Progesterone, prostaglandin E2, and hCG induce both conventional and plasmacytoid decidual dendritic cells to adopt tolerogenic phenotypes, which inhibit Th1 priming and promote Treg induction [3,33]. MERTK–Gas6 signalling eliminates apoptotic bodies and inhibits NF-κB activation, maintaining a non-sterile but non-detrimental inflammatory state. Macrophages polarise into M2-like states (CD163, CD206, MERTK), capable of efferocytosis, managing complements, and regenerating the matrix [71]. hCG indirectly enhances these myeloid processes by elevating IL-10 and diminishing TNF/IL-12. It may potentially perform this directly by associating cAMP with signalling in myeloid subsets. Complement is not a menace. It operates like a rheostat. Endothelial and trophoblast shields (CD46/CD55/CD59) safeguard against lytic damage, whereas local stromal C3 induction generates C3a–C3aR signals that influence leukocyte migration and trophoblast motility [12]. To protect the barrier, coordinated activation aligns with hCG-augmented junctional integrity (VE-cadherin/CD146) and promotes debris clearance and chemotaxis while limiting C5a-induced vascular permeability [10,12]. Chemokine axes are regulated to provide spatial corridors for invasion and repair that honour tissue boundaries. The axes for trophoblast guidance are CXCL12–CXCR4; for myeloid positioning, they are CCL2–CCR2 and CCL4–CCR5; and for vascular pathways, they are CX3CL1–CX3CR1 [33,67]. Galectin-9–TIM-3 checkpoints and adenosinergic signalling (CD39/CD73→A2A receptor) function as further regulatory mechanisms that hCG-conditioned settings may activate to facilitate remodelling without provoking cytotoxicity [72].

These findings collectively designate hCG as a primary immune regulator that harmonises cellular immunology and endocrine timing. It reprograms and reallocates uNK cells towards angiogenic and matrix functions, stabilises and expands Tregs through the IDO1 kynurenine AHR and IL-10/TGF-β pathways, and fosters complement homeostasis and DC/macrophage tolerogenesis [73]. When these immune effects are synchronised with endothelial junction development, stromal decidual survival/autophagy mechanisms, and epithelial adhesion preparedness, a complex receptive phenotype is established [8,10,73]. Their size is influenced by background variables, such as the ovarian stimulation environment, embryo stage, patient age, and combinations of maternal KIR and foetal HLA-C, as well as the dosage, concentration, dwell duration, and timing of embryo transfer. These aspects collectively elucidate the clinical signals contingent upon dosage, time, and the studied population in various research investigations [14,69]. This facilitates the use of intrauterine hCG with biomarkers. For instance, vascular (VE-cadherin/CD146) and stromal (ACTA2/NOTCH1/C3; Beclin1/LC3/p62 flux) metrics can be integrated with endometrial immunophenotyping (uNK density/topology via CD56^bright^ mapping; Treg FOXP3+ frequency; PD-L1/IDO1 DC signatures), soluble mediators in uterine fluid (IL-10/TGF-β, C3a), and peripheral surrogates (Treg proportion/function) to select candidates and enhance procedures.

## 5. Translational and Clinical Implications

To implement the mechanistic imprint of intrauterine hCG, it is essential to align various clinical signals with dose–timing–context relationships. Meta-analytic syntheses demonstrate that by controlling for volume and concentration, together with hormonally preparing the endometrium, the administration of roughly 500 IU at the time of transfer enhances implantation, clinical pregnancy, and, in multiple datasets, live delivery [6,7]. Conversely, despite intermittent improvements in biochemical markers and ongoing pregnancies, pooled randomised data for fresh embryo transfer do not provide any substantial benefits for live birth, implantation, or clinical pregnancy [31]. These discrepancies can be reconciled through a mechanistic perspective, demonstrating that the actions of hCG are meticulously aligned with the activation of immune tolerance programs, stromal survival/decidual initiation, endothelial junctional maturation, and epithelial adhesion competence [74]. Supraphysiologic hormones induce alterations in the endometrium and modify the tone of the microvascular and immunological systems; however, these transient, compartment-specific effects are misaligned and diminished, as shown in several fresh cycles [75]. Conversely, programmed frozen–thawed transfers diminish endocrine and inflammatory disturbances, facilitating concurrent uNK/Treg reprogramming, endothelial VE-cadherin/CD146 enhancement, stromal ACTA2/NOTCH1/C3 induction, and hCG-induced epithelial PI3K/Akt→eNOS activation (via miR-126-3p→PIK3R2), thereby promoting early invasion [10,12,62].

The structural variability of hCG introduces a further challenge. Various molecular variants, such as hyperglycosylated hCG and variably glycosylated isoforms, exhibit distinct bioactivity, receptor affinity, and downstream signalling properties. Irregular glycosylation patterns are linked to clinical disorders, including preeclampsia and impaired placentation. Hyperglycosylated hCG is prevalent in early pregnancy and has been connected with trophoblast invasion and placental growth. The structural differences may affect the immunomodulatory and endometrial effects of hCG, along with its luteotropic action, possibly modifying immune cell responsiveness, endothelial or stromal adaptability, and LHCGR signalling bias. Thus, understanding the molecular structure of hCG presents a crucial translational element, indicating that qualitative aspects of hCG exposure beyond simple dose and timing may influence implantation success and pregnancy results.

This paradigm yields two crucial determinants: receptor pharmacodynamics and intrauterine pharmacokinetics. The ligand is pharmacokinetically confined to the uterine cavity by a small-volume, high-concentration bolus, optimising dwell time at the luminal surface and microvasculature while limiting fast cervical and oviductal exit [76]. To enhance mucosal exposure to hCG, position the catheter near the fundal midline, limit the infusion rate to prevent reflux, manipulate the uterus delicately to minimise peristalsis, and provide a brief mucosal contact of 3–10 min prior to embryo transfer [77]. The trafficking and abundance of LHCGRs vary by compartment and cycle context; the dominance of cAMP/PKA, ERK, or PI3K/Akt pathways at any point is affected by β-arrestin scaffolding and the kinetics of receptor desensitisation and internalisation [78]. The interaction between steroids and LHCGR coordinates ESR1/PGR and LHCGRs during hormonally regulated cycles enhanced downstream signalling. In subsequent cycles, endometrial progression and modified vascular shear may interfere with receptor–effector coupling, diminishing the response to the same hCG pulse [10,12].

A further consequence is the use of phenotype-directed methodologies. Patients experiencing recurrent implantation failure frequently demonstrate immune dysregulation (marked by reduced Tregs and maladaptive uNK), impaired autophagic flux in stromal cells, attenuated LHCGR signalling, or compromised endothelial junctions, specifically the pathways that hCG can restore [3,8,10]. A ~500 IU bolus delivered minutes before transfer is mechanistically matched and has been correlated with enhanced implantation rates, clinical pregnancies, and, in some subgroups, live deliveries, notably in planned FET with blastocyst transfer [7,69]. In contrast, new cycles marked by a positive prognosis, maintained receptivity, and strong embryo-derived signalling may result in just a negligible marginal benefit [79]. Age (younger immune systems exhibit greater Treg expansions), endometrial thickness/vascularity, and embryo stage (D5 microenvironment aligns more effectively with hCG’s transient vascular/epithelial effects than D3) are likely additional factors that constrain the maximum response of endothelial and stromal cells [10,69]. Ultimately, when protocols align with mechanisms, both fidelity and safety increase. Augmenting NO-permissive microflow and VE-cadherin/CD146 adhesion mitigates the biological risk of oedema or hemorrhagic consequences, since hCG seemingly favours junctional maturation over neovascular development [8,10]. In addition to standardising dosage (approximately 500 IU), concentration (≥2 IU/μL), volume (≤500 μL), and pre-transfer timing, clinicians can enhance treatment specificity by integrating mechanistic readouts, epithelial (miR-126-3p/PIK3R2 targets), endothelial (VE-cadherin/CD146), stromal (ACTA2/NOTCH1/C3; Beclin1/LC3/p62), and immune (uNK topology, FOXP3+Tregs), to validate on-target biology in real time [7,8,10,36,73].

Patient stratification, operationalised through pre-transfer phenotyping of the endometrial compartments that hCG can reset, represents a pragmatic outcome. Women with RIF often have molecular problems that directly affect hCG-responsive axes [7]. These problems include weakened LHCGR signalling (lower receptor abundance/trafficking and weaker downstream PI3K–Akt–eNOS or ERK readouts), impaired proteostasis (low Beclin1/LC3 and high p62, which means that autophagic flux is blocked), and immune imbalance (suboptimal FOXP3+ T-regulatory tone and uNK profiles [8,73]). A ~500 IU intrauterine bolus has been consistently associated with increased implantation rates, clinical pregnancies, and, in several cohorts, live births, alongside peripheral Treg expansion as a measurable indicator of benefit in programmed frozen–thawed cycles [80]. This phenomenon is especially pronounced in younger patients undergoing blastocyst (D5) transfer, as the peri-implantation microenvironment is temporarily more congruent with the transient endothelial, epithelial, and immune effects of hCG [7,69]. However, there are fewer “correctable” defects in good-prognosis fresh cycles, which include intact receptivity, strong embryo-derived signalling, and supraphysiologic steroids that promote the endometrium and change shear/inflammatory tone; in these cases, pooled randomised analyses seem to limit incremental gain. These findings collectively endorse precision deployment: prioritise hormonally primed FET cycles, favour blastocyst-stage transfer, and select clinical scenarios exhibiting one or more identifiable actionable deficits, such as diminished competence of the LHCGR pathway (qPCR/IHC or downstream phospho-Akt/eNOS surrogates), junctional fragility (low VE-cadherin/CD146), stromal stress vulnerability (ACTA2/NOTCH1/C3 deficiency; Beclin1/LC3↓ with p62↑), or immune imbalance (low FOXP3+ Tregs, uNK topology skew). This aligns hCG’s pleiotropic, window-specific actions with the precise biological deficiencies most likely to result in implantation failure [3,8,10,12,69,73].

The therapeutic signal depends on both the length and effectiveness of the endometrial surface’s exposure to hCG, along with the hormone’s following effects upon binding. Consequently, protocol optimisation must be based on intrauterine pharmacokinetics and receptor biology [81]. Administering hCG in a minimal volume (≤500 μL) at an optimal concentration (≥2 IU/μL) shortly before transfer promotes mucosal bathing rather than rapid cervical reflux or isthmic spill. The recurrent efficacy threshold around ~500 IU likely indicates a receptor occupancy limit for multi-compartment LHCGR engagement, epithelial, stromal, and endothelial, when hCG is administered in this manner [7,82]. The distribution of drugs within the uterine cavity is predominantly regulated by mucociliary clearance, uterine peristalsis, and boundary layer diffusion through the glycocalyx [83]. A heightened concentration at a reduced volume amplifies the trans-epithelial gradient, consequently augmenting the likelihood that hCG will interact with LHCGRs on the apical epithelium and paracrine-accessible stromal/endothelial targets prior to dilution. The catheter should be positioned in the fundal mid-cavity. To avoid jetting, infusion must be conducted gently, with a brief dwell period and the elimination of air microbubbles. These precautions enhance local exposure and signal consistency. The details of the formulation are also significant. Contact time may be extended without inducing irritation using a pH/osmolality near physiological levels, utilising a carrier with little protein binding affinity, and selecting a low viscosity to mitigate shear-induced contractions. These kinetic factors clarify why endothelial junctional tightening (VE-cadherin/CD146), NO-permissive microflow, and epithelial surface adhesion remodelling are more reliably observed with dosing closer to transfer while concurrently initiating stromal survival and immune re-education programs with half-lives adequate to cover apposition and the initial influx of trophoblasts [8,10,12,73].

The pharmacodynamics of LHCGRs introduce additional mechanisms at the receptor level. The LHCGR is a class-A G protein-coupled receptor that primarily interacts with Gs, leading to the activation of cAMP and protein kinase A [84]. Biased signalling may occur when β-arrestin scaffolding and receptor trafficking direct ERK or PI3K/Akt–eNOS. This may influence adhesion, cytoskeletal tension, and endothelial shear responses variably, contingent upon the cell type [8]. The receptor reserve and desensitisation kinetics are dependent on the cycle stage; inconsistent steroid environments may separate ligand binding from downstream effectors, while luteal-phase steroids increase LHCGR expression and align ESR1/PGR with post-receptor transcriptional processes [85]. These characteristics promote planned FET scenarios, where receptor–effector coupling is more predictable, and a single, precisely timed bolus is preferred over split dosage to prevent rapid desensitisation [86]. A pharmacodynamic mismatch occurs when an identical hCG pulse interacts with a receptor environment that is either desensitised, temporally misaligned, or affected by complex second-messenger signalling. In novel cycles, supraphysiologic estradiol/progesterone often promotes endometrial advancement, modifies microvascular tone, and increases inflammatory activity [87]. Conversely, endocrine and inflammatory baselines exhibit greater stability in hormonally primed FET, facilitating a brief intrauterine exposure that engenders coherent multi-axis activation, particularly during the transfer window encompassing epithelial PI3K/Akt→eNOS via miR-126-3p→PIK3R2, endothelial VE-cadherin/CD146 stabilisation, stromal ACTA2/NOTCH1/C3 induction, and recalibration of uNK/Treg [8,10,12].

This framework results in beneficial enhancements to protocols. Refrain from uterotonic stimuli, including excessive manipulation, that may accelerate clearance. Insert the catheter into the upper mid-cavity and provide the infusion gradually. Permit a short interval prior to embryo loading to reduce quick washout, and sustain about 500 IU at a concentration of no less than 2 IU/μL in a volume not exceeding 500 μL. When feasible, synchronise blastocyst (D5) transfer in planned FET, ensuring that the peri-implantation milieu is temporally congruent with the acute vascular/epithelial responses and subacute stromal/immune mechanisms induced by hCG [7,12,69,82]. Clinics can verify that the selected dose–volume–timing combination activates the desired pathways, reduces inter-centre variability, and enables further optimisation by incorporating on-target pharmacodynamic evaluations, such as endothelial VE-cadherin/CD146 immunophenotyping, stromal ACTA2/NOTCH1/C3 induction, epithelial p-Akt/eNOS or miR-126-3p/PIK3R2 signatures, luminal fluid analysis for hCG concentration time, and immune alterations in uNK topology or FOXP3+ Tregs [12,69].

Having current safety and viability data is reassuring. Following standard embryo transfer protocols has not consistently correlated intrauterine hCG with heightened risks of miscarriage, ectopic pregnancy, or multiple gestations compared to predicted baselines in randomised trials or cohort studies [7,82,88]. The hCG-associated PI3K/Akt–eNOS activation fosters a shear-stable, anti-inflammatory endothelium phenotype that exhibits reduced vulnerability to barrier failure [89]. Nevertheless, the in vivo endometrial observations, specifically the targeted augmentation of endothelial adherens junctions (VE-cadherin/CD146), in the absence of a corresponding rise in angiogenic factors, such as VEGFR1/2, contradict the hypothesis of pro-edematous neovascular leakage from a mechanistic standpoint. The clinical observation that cramping, bleeding, or vasovagal reactions are infrequent and typically self-limiting when employing an atraumatic technique and avoiding excessive distension of the uterine cavity aligns with these physiologically significant safety indicators [90].

A solution must be carefully planned and carried out in order to be operationally feasible. Recombinant hCG formulations contain fewer immunogenic contaminants than urine-derived products. Solutions should be made with a physiological pH/osmolality and low-protein binding to obtain the most interaction with the mucosa while causing the least irritation. It is very important to perform procedures in a sterile way, double-check the dosage and concentration, and keep particles and pyrogens from getting into the mix [91]. Gradual, low-volume administration extends mucosal exposure to the medication, reduces jetting and reflux, and limits mechanoreceptor stimulation that could trigger contractions through prostaglandins. Using strict single-use disposables and keeping the area clean may lower the chance of getting an infection. Putting the catheter into the upper mid-cavity and checking with ultrasound helps stop endocervical pooling or localised over-pressure. Facilities that standardise documentation (pre-/post-instillation cavity evaluation), delivery (infusion rate, dwell duration), and compounding (lot traceability, beyond-use period) exhibit commendable procedural repeatability and minimal disruption of transfer logistics [7,36].

Risk management that takes the situation into account is better than risk management that makes people afraid. To avert ascending infection, patients with endometritis, a persistent cervicovaginal infection, or recent uterine instrumentation should postpone procedures until the condition is resolved [92]. Important warning signs are severe irritation of the uterus, severe cervical stenosis that needs aggressive dilation, or too much fluid building up in the uterus. These disorders may make it harder to place the embryo and raise the risk of it being ejected [93]. Even though hCG’s main effect on blood vessels is junctional maturation instead of angiogenic proliferation, which makes bleeding less likely, it is still a good idea to be careful and limit contact with the fundus. Even though hCG’s immunomodulatory effects (Treg expansion, uNK re-patterning) make pro-inflammatory flares less likely, doctors should still watch for unusual pain or fever and make sure that patients can easily reach them and obtain clear instructions after the procedure [69,73]. Finally, timely instillation within the right time frame lowers the risk of moving the embryo and the need for extra surgeries, which keeps the good benefit–risk profile that has been seen so far. Synchronised time for transfer functions as both a safety measure and an efficacy determinant [7,8,10,82].

By identifying deficient endometrial axes that are responsive to hCG, mechanism-anchored biomarkers can convert intrauterine hCG from a universal adjunct into a stratified intervention. In practice, this means making a pre-transfer phenotyping panel that uses useful tests to sample different compartments [94]. Immunohistochemistry or multiplex immunofluorescence on a Pipelle biopsy reveals ACTA2 (α-SMA), NOTCH1, and C3 on the epithelial/stromal side, indicating early decidualisation and survival tone [12]. Quantitative PCR or targeted RNA panels can evaluate HOXA10/ITGB3/LIF and FOXO1 (ERA-adjacent signatures). For the vascular interface, you can obtain a cohesion index that matches the in vivo effects of hCG on endothelial junctions by immunophenotyping VE-cadherin (CD144) and CD146 (MCAM) on endometrial microvessels (digital image analysis for junctional continuity) [10]. In addition to checking for PD-L1/IDO1 expression on decidual dendritic cells to see if they are presenting tolerogenic antigens, you can also use flow cytometry or CyTOF on endometrial aspirates to check for FOXP3+ Treg frequency/function and uNK density/topology (CD56brightCD16) [69,73]. When a biopsy is not feasible, uterine fluid, referred to as the “endometrial secretome,” serves as a minimally invasive matrix for VEGF, CCL4, HGF, IL-10/TGF-β, and emerging cargo, such as miR-126-3p, in cell-free RNA or extracellular vesicles. This represents an upstream epithelial signal that mechanistically connects to PIK3R2→PI3K/Akt→eNOS activation [8,62]. Imaging adjuncts, such as 3D power Doppler subendometrial vascular indices, can offer a macroscopic correlate of microvascular poise without supplanting tissue-level markers. Pre-analytical control is necessary to keep inter-lab drift to a minimum. This means making sure that the cycle day/progesterone exposure, sampling site, cold chain, RNase inhibitors for miRNA work, and gating/thresholds for immune phenotyping are all the same [95].

This enables the implementation of a two-step algorithm. Step 1: Phenotype. Use lab-normalised z-scores or predetermined cut-offs to put the patient into one or more deficit classes [96]. For example, for immunological deficits, use low FOXP3^+^ Tregs, malpositioned/low-density uNK, and high APC activation with low PD-L1/IDO1; for endothelial deficits, use low VE-cadherin/CD146 continuity; and for epithelial/stromal deficits, use low HOXA10/ITGB3/FOXO1, low ACTA2/NOTCH1/C3 [97]. Step 2: Targeted Deployment. Give about 500 IU of hCG in ≤500 μL at ≥2 IU/μL minutes before the transfer. This is the best time to take advantage of the temporary effects on epithelial, endothelial, and immune cells in phenotypes with LHCGR insufficiency, weak junctions, or uNK/Treg disequilibrium [7,10,73,82]. A simulated “hCG challenge” cycle, involving uterine fluid sampling at 15–60 min to validate anticipated changes, such as an increase in miR-126-3p, VEGF/CCL4, or junctional surrogates or peri-procedural micro-aspiration for p-Akt/eNOS in epithelial cells, can be utilised as an on-target pharmacodynamic assessment in research contexts. This can be accomplished before the protocol is executed in the treatment cycle [8,10,62]. The co-primary endpoints combine live birth with mechanistic PD (VE-cadherin/CD146 gain, ACTA2/NOTCH1/C3 induction, Treg/uNK rebalancing). After that, biomarker-enriched, adaptive trials should randomise by dose (250/500/1000 IU), volume (200–500 μL), and timing (5/30/60 min pre-ET). This design will operationalise hCG as a mechanism-anchored, patient-selected adjunct rather than an indiscriminate add-on by defining which deficit profiles receive the greatest absolute benefit and optimising dose–dwell–timing parameters [10,12,73].

## 6. Comparison with Other Endometrial Modulation Strategies

The principal axis employed by endometrial “priming” strategies can be categorised into immune conditioning to improve tolerance, matrix/adhesion alterations to promote capture, exogenous growth factor infusion to enhance trophicity, or controlled injury to trigger an inflammatory reset [98]. Intrauterine hCG is fundamentally different from a unidimensional nudge, as it is an embryo-mimetic ligand that engages with a particular GPCR (LHCGR) co-expressed in the epithelium, stroma, and endothelium. This enables the simultaneous activation of numerous compartments [2]. A brief intrauterine pulse can (i) modify the adhesive properties of epithelial cells by rapidly altering the membrane and cytoskeleton, (ii) enhance the rigidity of endothelial junctions and facilitate microflow through eNOS phosphorylation, and (iii) initiate early stromal survival/decidual programs (ACTA2, NOTCH1, C3) that persist beyond the immediate contact duration. The LHCGR not only interacts with Gs→cAMP/PKA but also recruits β-arrestin scaffolds that channel signals to ERK and PI3K/Akt–eNOS pathways, which are particular to the cell type [99]. The ensuing paracrine cascades secretome modification and immunological predisposition towards uNK/Treg-mediated tolerance are biologically programmed rather than arbitrary, since these pathways are inherently connected to an innate prenatal signal [73]. Injury-centric methodologies depend on the erratic timing and magnitude of IL-1β/IL-6/TNF surges. Growth factor mixtures, like PRP, exhibit variability across batches and lack a unified cognate receptor [100]. Pure adhesion enhancement transpires exclusively at the point of contact, failing to condition the immune milieu or vascular framework, and immune-targeted adjuncts frequently modify tolerance without rectifying endothelial or epithelial dynamics [101]. Thus, the receptor-bound, temporally regulated pharmacology of hCG provides extensive axis coverage, epithelial, stromal, endothelial, and immune, within a limited, clinically feasible timeframe, creating a coherent mechanistic link between a singular, precisely timed intrauterine ligand and the necessary coordinated tissue condition for implantation [8,10,102].

PRP comprises a combination of platelets, including PDGF, TGF-β, VEGF, IGF, and chemokines. It may stimulate SMAD2/3, PI3K/Akt, and ERK in epithelial and stromal cells, subsequently influencing angiogenesis, fibroblast motility, and the equilibrium of MMP and TIMP [103]. Nonetheless, its efficacy is intricately associated with preparatory variables. The diffusion radius within the uterine cavity and the release kinetics from α-granules, whether burst or sustained, are affected by fibrin architecture, activation methods (CaCl_2_, thrombin, freeze–thaw), LR-PRP versus LP-PRP composition, and the centrifugation protocol [104]. These levers may modify the flow next to the wall and the epithelial ciliary clearance by altering the inflammatory tone (LR-PRP typically elevates IL-1β/IL-8), the local rheology, and the deposition of ECM via TGF-β-mediated pathways [105]. PRP has several molecular parallels with components of the hCG cascade, particularly in relation to VEGF-associated vascular modulation and stromal remodelling. Nonetheless, it lacks a singular cognate receptor and varies across batches, complicating its synchronisation with the window of intervention and reproducibility among patients [106]. Conversely, hCG activates the LHCGR to emit a GPCR-biased signal that can be temporally stabilised and repeatedly retrieved; for instance, the upregulation of VE-cadherin/CD146 in the endothelium, the induction of ACTA2/NOTCH1/C3 in the stroma, and the activation of miR-126-3p→PIK3R2→PI3K/Akt→eNOS in the epithelium. This facilitates the verification of PD on target and precise synchronisation with the transfer [2,8,10,12]. In phenotypes marked by decidual stress (vulnerability to proteostasis) or junctional fragility (permeable microvasculature), hCG offers a distinct mechanism that PRP’s trophic range cannot reliably replicate. A pragmatic allocation of tasks is as follows. hCG serves as the more appropriately timed intervention when addressing adhesion–vascular–immune integration during the implantation window, while PRP may function as a precursor enhancer when the issue pertains to cellularity or perfusion in cases of thin, hypotrophic endometrium [3]. Sequential protocols incorporating various PD checkpoints (PRP: thickness/3D-Doppler/VEGF in uterine fluid; hCG: VE-cadherin/CD146, epithelial p-Akt/eNOS, and immune re-patterning) can reduce redundancy while optimising the efficacy of each agent when utilised in conjunction [2,8,12].

Activation of the JAK/STAT3, PI3K/Akt, and MAPK pathways in haematopoietic and particular endometrial cells by G-CSF signals via CSF3R redirects neutrophil trafficking and skews dendritic/macrophage polarisation towards tissue-constructive states [107]. Moreover, VEGF/MMP pathways are subsequently engaged, potentially augmenting cellularity and perfusion within a slender functional layer. This results in two partially distinct effects: (i) substrate development, which enhances the endometrial thickness and the underlying vascular indices by attracting endothelial progenitor cells, leading to stromal proliferation and matrix alteration; and (ii) myeloid modulation, occurring when dendritic cells and macrophages adopt PD-L1/IDO1-positive, IL-10-rich phenotypes that indirectly promote Treg expansion and diminish Th1/Th17 activity [108]. The manifestation of these advantages is gradual and contingent upon the substrate. The myeloid axis alone does not enhance decidual stress resistance, endothelial adhesion preparedness, or endothelial junctional integrity [109]. Consequently, clinical indicators in thin endometrium and repeated implantation failure remain ambiguous. This is the locus of collaboration between hCG and G-CSF. G-CSF facilitates the development and recruitment of cells, while hCG stabilises multi-compartment preparedness at the critical period by reinforcing VE-cadherin/CD146 junctions, altering the distribution of uNK/Tregs, and initiating early decidual and survival mechanisms [10,73,102]. It is logical to adhere to a biomarker-guided protocol. Administer G-CSF earlier during endometrial preparation to restore myeloid tone and cellularity (monitor thickness, 3D power Doppler, and DC/macrophage PD-L1/IDO1), followed by pre-transfer hCG to align immune tolerance with epithelial/vascular priming (assess on-target PD using VE-cadherin/CD146 gain and Treg/uNK rebalancing). This choreography encompasses the whole process, from substrate adequacy to window-specific receptivity. It reduces duplication and leverages the distinct strengths of each agent’s mechanism [10,73,102].

The objective of endometrial scratching is to “reset” receptivity by initiating a brief, self-limiting wound healing process that encompasses the release of danger signals (ATP, HMGB1), the activation of toll-like receptors 2 and 4, the activation of NF-κB, and an increase in cytokines and matrix metalloproteinases (IL-1β, IL-6, TNF, MMP-2/-9) [110]. A characteristic of the hCG-induced secretome identified in 3D human endometrium (CCL4/FGF2/IL-1β/IL-6/IL-17) exhibits similarities. However, the underlying input is fundamentally distinct, originating from a mechanical DAMP-driven program whose magnitude, spatial distribution, and kinetics present significant standardisation challenges [2,62]. Scratching may recruit myeloid cells that establish a permissive matrix, therefore temporarily enhancing stromal remodelling and epithelial plasticity via EMT-like signalling and protease activity [111]. In individuals with preexisting illness, the same mechanisms, excess IL-1β/TNF, heightened MMP activity, or increased contractility from prostaglandins, can become excessive, leading to premature inflammation, epithelial denudation, or an inadequate luminal barrier upon the embryo’s arrival [112]. The vascular (VE-cadherin/CD146) and survival/autophagy pathways, often constrained in IVF, have not been addressed because of the non-receptor and non-selective nature of the trigger, which fails to inherently enhance endothelial adherens junctions or reinstate stromal proteostasis [10,12].

In contrast, hCG elicits a mild inflammatory response through receptor-mediated paracrine modulation. It fortifies endothelial junctions, increases specific cytokines without causing significant tissue damage, enhances nitric oxide-permissive microflow, and activates early decidual/survival pathways and autophagic flux, which remain unaffected by biological scratching [8,10]. When considering scratch, mechanical phasing is crucial. Executing it in the prior cycle, instead of peri-transfer, reduces the likelihood of simultaneous new damage during apposition and aids in mitigating inflammatory peaks while maintaining potential reprogramming. A diminutive endometrial aspirate or uterine fluid specimen collected 24–72 h post-scratch may quantify IL-1β, IL-6, TNF, and MMPs (or a concise transcript panel) to verify that the body is progressing towards a receptive setpoint. If the inflammatory indicators remain elevated, it is inadvisable to proceed without implementing modifications. Monitoring during the operation helps mitigate the risk of utilisation due to varying reactions [113]. Active endometritis or cervicovaginal infection, significant uterine irritability, and phenotypes defined by endothelial cohesion or stromal stress resilience are contraindications. In these instances, hCG has a direct influence on the mechanism, whereas scrape introduces interference [114].

Subsequently, there exists a pragmatic allocation of tasks predicated on biomarkers. If the primary issue is a low inflammatory tone and inadequate myeloid recruitment, despite otherwise favourable junctional and decidual characteristics, a strategically timed scratch may elicit the necessary inflammatory response. This is best substantiated by a transient elevation in cytokines that diminishes before transfer [62,102]. If the issues pertain to an imbalance in immunological tolerance, insufficient decidual survival or autophagy, or compromised junctions, receptor-level hCG priming is the preferable option. Sequential designs (initiating anew in a prior cycle with PD confirmation of resolution, succeeded by pre-transfer hCG in the treatment cycle) can diminish redundancy and optimise the advantages of each instrument while maintaining the precise, time-sensitive signal that hCG offers for centres exploring combination protocols [114].

Hyaluronan-enriched transfer media function at the point of contact, where the transition from early tethering to strong adhesion is affected by ligand availability and fluid dynamics. High-molecular-weight hyaluronan stimulates CD44 and RHAMM on the luminal epithelium and trophoblast, facilitating integrin clustering (αvβ3/β1) and the rapid formation of focal adhesion scaffolds (FAK/Src–talin–vinculin) [115]. It performs this by regulating cortical actin via ERM and Rho family switches, using Rac1 for edge stability and modulating RhoA to prevent junctional disruption. From a biophysical standpoint, increasing the viscosity of hyaluronan near the wall improves capture probability and the transition from L-selectin to integrin, performing this without necessitating excessive force [116]. This is achieved by minimising slip, extending residency duration, and alleviating disruptive microvortices at the catheter–endometrium contact. Hyaluronan alters the glycocalyx by temporarily displacing strongly anti-adhesive mucins to expose osteopontin–integrin contacts. At therapeutic quantities, hyaluronan demonstrates immunoquiescence, hence reducing inadvertent cytokine elevations [102]. These moment-specific effects inherently enhance hCG, which functions upstream to reinforce VE-cadherin/CD146 at the microvasculature, augment decidual survival mechanisms in the stroma, elevate integrin preparedness in the epithelium, and skew uNK/Treg circuits towards tolerance prior to the embryo’s arrival [2,10,12]. Incorporating hyaluronan into pre-transfer hCG priming bridges the disparity between the biology of competent tissue and the physics of apposition in patients with normal HOXA10/ITGB3/LIF and adequate endothelial/immune indices, but it experiences adhesion-stage failure.

hCG induces an immunological bias in the body that aligns with the process of implantation. Immune-directed adjuncts, conversely, aim to enhance the body’s tolerance by targeting antigen-presenting cells and lymphoid subsets [73]. It not only enhances PD-L1/IDO1-positive dendritic/macrophage programs that inhibit Th1/Th17 activity and facilitate spiral artery remodelling but also elevates FOXP3^+^ Tregs and transforms uNK cells into pro-angiogenic, matrix-modulating phenotypes with little cytotoxicity [3,33,97]. Therefore, hCG should be the principal treatment, coordinated with transfer, for patients with clear immune dysregulation, including decreased Tregs, improperly localised or low-density CD56^bright^CD16^−^ uNK cells, or lowered PD-L1/IDO1 expression on decidual dendritic cells [69]. To prevent counter-phasic or redundant effects and excessive suppression that might hinder trophoblast-mediated vascular remodelling, any further immune intervention (e.g., procedures that condition PBMCs or modify APC checkpoints) must depend on on-target immunological PD. Examples of these immunological strategies include the activation of dendritic cell PD-L1/IDO1, improved spatial measures of uterine natural killer cells, a pro-angiogenic secretome, and the restoration of the Treg/Th17 ratio [69,73,97]. A biomarker-driven sequence is advantageous since it maintains the precision and temporal advantages of hCG while modulating the intensity to align with the patient’s immunological profile. This entails validating the immunological deficiency, using pre-transfer hCG to recalibrate the uNK/Treg axis, and only introducing a further immune intervention if PD remains poor [7].

Generally, one should not choose a technique predicated on aggregation. It should be predicated on what is absent instead. When microvascular cohesion and shear stability are impaired, hCG’s junctional reinforcement and NO-permissive flow immediately mitigate the vascular bottleneck [10]. In cases when decidual survival/proteostasis is compromised, hCG’s stimulation of ACTA2/NOTCH1/C3 and the reestablishment of Beclin1/LC3/p62 equilibrium address the fundamental biological issues [8]. If the endometrium is thin, a G-CSF or PRP build-up stage may be necessary, followed by hCG to synchronise the final window. If adhesion is the primary issue, using hyaluronan over hCG may be beneficial. To convert empirical enhancements into a mechanism-based toolkit focused on hCG as the embryo-mimetic, time-sensitive modulator, a biomarker framework that includes epithelial/stromal signatures, endothelial cohesion indices, and immune tolerance metrics should govern these evaluations [10,12,62,73].

## 7. Discussion

The mechanistic routes outlined above should be seen as linked layers of a singular peri-implantation network, rather than as distinct or contradictory explanations, to facilitate the integration of concepts. hCG regulates many facets of endometrial receptivity, including immunological tolerance, stromal decidual survival, endothelial junctional stability, and epithelial adhesion competence, within a limited temporal context. The variety in clinical outcomes after intrauterine hCG supplementation may be elucidated via molecular diversity by conceptualising these pathways as an interconnected system.

Our narrative synthesis endorses a coherent, mechanism-driven paradigm whereby intrauterine hCG acts as an embryo-mimetic, multi-compartment regulator of endometrial receptivity. Preclinical and translational research demonstrates that hCG activates luteinising hormone/hCG receptors (LHCGRs) on epithelial, stromal, and endothelial cells, coordinating four interconnected axes: immune tolerance re-patterning, characterised by the proliferation of FOXP3^+^ regulatory T cells and the reorientation of uNK cells towards pro-angiogenic, matrix-modulating states; endothelial junctional maturation, indicated by selective increases in VE-cadherin (CD144) and CD146 (MCAM) without the upregulation of VEGFR1/2; and stromal decidualisation–survival/proteostasis, identified by the induction of ACTA2, NOTCH1, and C3, along with the restoration of autophagic flux (Beclin1/LC3/p62). These consistent mechanistic markers clarify a biological route that explains the diversity of therapeutic effects contingent upon context, time, and dosage. Administering about 500 IU of hCG a few minutes to an hour before transfer at a regulated volume/concentration yields the most consistent signals in hormonally programmed frozen–thawed cycles. Nonetheless, recent transfers exhibiting endometrial progress, increased shear, and inflammatory noise demonstrate minimal impact in aggregated randomised data [7,82]. Intrauterine hCG is associated with improved implantation and clinical pregnancy rates, and in specific cohorts, live birth rates, as well as peripheral Treg expansion in RIF, where LHCGR functionality, stromal proteostasis, endothelial integrity, and immune balance are often compromised. This is particularly applicable to younger individuals undergoing a blastocyst transfer [7,69]. The vascular phenotype characterised by junctional reinforcement without angiogenic proliferation displays a biologically anti-edematous response under NO-permissive microflow, and clinical studies do not consistently indicate increases in miscarriage, ectopic pregnancy, or multiple gestation [7,10,82]. These safety indicators are comforting. Table 3 presents controlled clinical trials and meta-analyses of intrauterine hCG categorised by cycle type, dosage interval, exposure parameters, embryo stage, results, and safety. This elucidates the influence of environment and time on clinical outcomes.

Table 3 demonstrates a continuous pattern dependent upon context and time. Clinical trials indicate no variation in clinical outcomes when hCG is administered 24 to 72 h before embryo transfer or in subsequent cycles [117,118,119]. This corresponds with a pharmacodynamic inconsistency between the dosage interval and temporary epithelial/vascular openings. Research increasingly demonstrates improvements in implantation and clinical pregnancy rates, along with live birth rates in specific cohorts, in RIF/programmed FET, particularly with micro-volume, high-concentration exposure immediately prior to embryo transfer (≤500 μL; ≥2 IU/μL; ~500 IU). Meta-analytic validation improved parameters and established safety (Luo; Gao) [7,82]. Human mechanistic investigations validate immunological tolerance (Treg/uNK bias), stromal survival/decidual initiation (ACTA2/NOTCH1/C3), and junctional maturation (VE-cadherin/CD146 enhancement). This explains why administering a peri-transfer bolus in planned cycles may provide therapeutic advantages, but dosing the previous day does not [10,12].

The receptor-bound pharmacology and temporally controllable footprint of hCG distinguish it from other techniques of endometrial “priming.” G-CSF may generate substrate and instruct myeloid compartments; however, it does not regulate the timing of adhesion, vascular, or immunological coupling during transfer. An endometrial scrape lacks specificity and is difficult to classify. Hyaluronan functions only at apposition, whereas PRP offers diverse trophic input but lacks a singular corresponding receptor and is contingent upon its preparation [121]. During the implantation window, hCG orchestrates epithelial adhesion, endothelial cohesiveness, stromal viability, and immunological tolerance via the activation of LHCGRs across several compartments. In cases when adhesion mechanics are ineffective, hyaluronan may be applied externally [2,8,10,12]. Ultimately, the identical mechanistic map provides a feasible approach for precise implementation. Characterise the immune interface and endometrium (HOXA10/ITGB3/LIF; ACTA2/NOTCH1/C3; VE-cadherin/CD146; FOXP3+ Tregs/uNK topology/PD-L1–IDO1), which is succeeded by a rigorous pre-transfer of approximately 500 IU of hCG in profiles exhibiting uNK/Treg imbalance, junctional instability, or LHCGR deficiency. When possible, accurate pharmacodynamic evaluations are included [10,12,73]. Utilising this approach, intrauterine hCG transitions from a general adjunct to a patient-selected IVF intervention grounded on a particular mechanism.

Recent mechanistic developments have included epigenetic and paracrine aspects into fast GPCR signalling, therefore enhancing the understanding of hCG’s role in the implantation cascade [60]. Brief exposure to hCG in decidualised human stromal cells modifies the expression of chromatin modifiers (TET1/2/3, HDAC1, EED) and increases H3K27 acetylation at essential receptivity/decidual enhancers (FOXO1, HOXA10, HAND2). This pattern is anticipated to stabilise progesterone-responsive transcriptional loops and activate lineage-defining enhancers [60]. Concurrently, hCG modifies the composition of EVs by incorporating certain miRNAs (such as miR-340-3p, miR-663a, miR-766-5p, miR-3138, and miR-3180-5p) and proteins associated with implantation and pregnancy (including IGFBP1/7, PAPP-A, MMP-2, and STC-1/2) [122]. This enables the stroma to transmit signals to the epithelium, endothelium, and resident leukocytes [60]. hCG not only activates transient second messengers but also modifies chromatin accessibility and transmits signals that extend receptivity cues beyond the initial target cell, persisting throughout the early post-transfer period.

Alongside this stromal remodelling, the epithelium functions as an active conduit. hCG generated from embryos increases the expression of cPLA2, COX-1/2, and AKR1C3 in luminal epithelial cells. This enhances the synthesis of PGF_2_α, subsequently prompting ADAM17 to release EREG/HB-EGF. This influences EGFR signalling, which promotes stromal decidualisation markers, such as IGFBP1 [123]. This delineates a prostaglandin–EGFR axis that precedes traditional decidual gene programs and offers a biochemical justification for the effects of precisely timed hCG administration. EGFR ligands and epithelial PGF_2_α may prime the underlying stroma within minutes as the embryo approaches the surface [94]. Nitric oxide is concurrently transported to the interface by known epithelial routes, particularly through the suppression of PIK3R2 (p85β) by miR-126-3p, which mitigates the inhibition on PI3K p110α→Akt, leading to the phosphorylation of eNOS Ser1177 [8]. The absence of sGC activation leads to cGMP and PKG promotion, facilitating lubricated apposition and altering the micro-shear experienced by the blastocyst during settling by rapidly reducing junctional tension and modifying cortical actomyosin.

The vascular plane of hCG does not proliferate; rather, it develops at junctions. The selective expression of VE-cadherin (CD144) and MCAM (CD146) on endometrial endothelium, without concurrent modifications to VEGFR1/2, indicates improved lateral cohesion and stabilisation of adherens complexes (VE-cadherin–β-catenin–p120), consequently reducing paracellular leakage while maintaining regulated permeability [10]. Nitric oxide produced by the epithelial PI3K/Akt–eNOS axis likely functions synergistically by increasing barrier function via cGMP-PKG and reducing Src-dependent VE-cadherin phosphorylation events that would otherwise compromise junction stability [8,10]. The final outcome is a microvasculature that remains resilient under stress and facilitates the efficient exchange of cytokines and metabolites without breaching the interface. This configuration is ideal for the initial, non-invasive infiltration of trophoblasts and precise alignment.

Intrauterine hCG activates an early survival/decidual mechanism in the stroma, which is facilitated by C3, NOTCH1, and ACTA2 (α-SMA) [12]. C3 introduces a modifiable complement tone that facilitates clearance and chemotaxis while minimising cytotoxicity. NOTCH1 influences stromal cell fate determination and vascular–stromal interactions. ACTA2 enhances the subepithelial/perivascular myofibroblast network, hence increasing tissue rigidity in areas requiring regulated force transfer. The transcriptional alterations, together with improved proteostasis (shown by reduced p62 and increased Beclin1/LC3), and the activation of ERK1/2 and mTOR [8] elevate the stroma’s resilience to oxidative and metabolic stress during implantation. The result is a dance consisting of four sections: initial transcriptional waves (2–6 h) in epithelial, endothelial, and stromal targets; extended (24–72 h) epigenetic and extracellular vesicle-mediated reinforcement that sustains receptivity cues during apposition and early invasion; rapid (seconds–minutes) GPCR/NO and junctional modifications; and intermediate (10–60 min) ADAM17→EREG/HB-EGF shedding and EGFR signalling [10,12,120]. To convert the mechanism into a replicable practice, Table 4 delineates a standardised intrauterine hCG “exposure recipe” aligned with mechanistic evidence and justification. This includes the dose, concentration, volume, timing, catheter technique, and built-in pharmacodynamic checkpoints.

The typical exposure protocol (~500 IU at ≥2 IU/μL in ≤500 μL, delivered minutes before ET with meticulous catheter technique and a short dwell time) synchronises hCG’s first epithelial and endothelial phases with embryo implantation [10,12,73,120,123]. This initiates mechanisms for stromal survival and immunological tolerance that last until early invasion. The basis of this practice in essential PD evaluations (including uterine-fluid EREG/HB-EGF/NOx, epithelial p-Akt/p-eNOS, VE-cadherin/CD146 continuity, ACTA2/NOTCH1/C3, FOXP3+ Tregs) clarifies why programmed FET with D5 blastocysts constitutes the most responsive environment, thus transforming a mechanistic hypothesis into a testable clinical quality control [35,69].

The clinical literature clearly distinguishes between time and cycle context within this mechanistic paradigm. A quasi-experimental RCT administering 500 or 1000 IU one day before blastocyst transfer showed no improvement compared to the lack of hCG in fresh stimulation cycles [118]. This pattern transcends mere negativity; it precisely aligns with expectations of a pharmacodynamic mismatch. Recent cycles often exhibit supraphysiologic concentrations of oestradiol and progesterone, varying amounts of hCG trigger exposure, and endometrial progression [125]. These parameters may distinguish the LHCGR from its downstream effectors, alter the implantation window, and modify the immunological profile and microvascular shear at the interface [126]. In addition to neglecting the brief epithelial and endothelial windows (minutes to hours) that facilitate adhesion competence and junctional tightness, administering hCG 24 h prior to ET may diminish receptor sensitivity before embryo implantation. Despite the initial elevation of biochemical signals, they fail to translate into stable clinical outcomes due to the hormone’s presence in a tissue where the phase and receptor state are misaligned [10].

The gap is important, even in frozen procedures; a randomised research providing 500 IU between 48 and 72 h prior to embryo transfer showed no benefit [119]. This emphasises that the physiologically actionable timeframe is not days but rather minutes to an hour prior to transfer. Over extended durations, the ambient endocrine milieu has either supplanted or degraded the acute outputs upon which humans depend. This encompasses the first phase of stromal survival/decidual signalling, VE-cadherin/CD146 junctional fortification, ADAM17-mediated shedding of EREG/HB-EGF, and the activation of epithelial PI3K/Akt leading to eNOS activation [127]. Practical considerations enhance this further. Administering higher volumes at reduced concentrations accelerates reflux and dilution, hence decreasing the duration of mucosal retention and diminishing receptor activation. Smaller-volume, higher-concentration boluses at the ET enhance compartment-spanning activation and interaction with the boundary layer at the embryo’s endometrial interface.

Meta-analytic analysis only examining fresh transfers supports the timing/context hypothesis. Despite rare elevations in biochemical or continuing pregnancies, combined randomised controlled trials do not demonstrate any increase in live birth or clinical pregnancy rates [117]. The most economical interpretation is “signal without endpoint,” suggesting that although specific early events (such as apposition or initial trophoblast signalling) are facilitated, the benefit wanes before clinical milestones due to the asynchronous activation of vascular, stromal, and immune programs at the time of embryo–endometrium interaction [128]. The introduction of noise to receptor occupancy and post-receptor bias (cAMP/PKA vs. ERK versus PI3K/Akt–eNOS), together with variations in dosage, concentration, volume, duration, embryonic stage at transfer, luteal support, and the presence of systemic hCG, complicates the identification of any genuine impact [129]. Thus, methods that streamline the dose–exposure–transfer process during the peri-transfer minutes and control volume/concentration are more likely to translate mechanistic improvements into lasting therapeutic effects. These facts do not refute the process; rather, they suggest that misaligned biology obscures it.

Conversely, evidence examines RIF and programmable FET settings, whereby one may regulate the endocrine tone and timing. In these contexts, the endometrium often exhibits actionable deficiencies that correspond to hCG-responsive pathways [130]. The proliferation of peripheral Tregs, a pharmacologic marker of tolerance programming, correlated with heightened implantation rates, clinical pregnancies, and live babies in a prospective cohort of RIF/FET patients. The effects were especially significant in younger individuals and during blastocyst transfers [69]. A randomised trial focused on RIF/FET showed that administering a micro-volume (about 40 μL) of hCG around three minutes before ET enhanced implantation and clinical pregnancy rates. This research highlighted two viable strategies: small volume/high concentration to enhance mucosal retention and ultrashort lead time to capitalise on hCG’s transient epithelial and endothelial possibilities [120]. The meta-analytic synthesis validates these themes. In RIF, live birth rates improve but are inadequately powered in combined RIF datasets; clinical pregnancy and implantation rates consistently increase when protocols are standardised to approximately 500 IU, ≤500 μL volume, and ≥2 IU/μL concentration, with safety comparable to controls [7].

From a mechanical perspective, these advantages, which are valid only under certain circumstances, seem logical. LHCGR insufficiency, stromal proteostasis stress (shown by reduced Beclin1/LC3 levels and p62 accumulation), junctional fragility in the microvasculature, and immunological dysregulation (demonstrated by maladaptive uNK topology and decreased FOXP3+ Tregs) are common in RIF endometria. Intrauterine hCG may correct these defects between minutes and hours [31]. This includes epithelial miR-126-3p→PIK3R2→PI3K/Akt→eNOS for adhesion preparedness, endothelial VE-cadherin/CD146 fortification for barrier integrity, stromal ACTA2/NOTCH1/C3 activation with the reestablishment of autophagic flux for survival/decidual onset, and immune reconfiguration towards a Treg/uNK-supported, tolerogenic environment [8,10,12]. Recent clarifications concerning the unequal impacts of peri-transfer, micro-volume dosing in programmed cycles pertain to the epigenetic priming of decidual enhancers and the reprogramming of extracellular vesicle cargo within stromal cells, alongside an epithelial PGF_2_α→ADAM17→EREG/HB-EGF relay that expedites stromal decidual signals [60,123]. A brief hCG pulse may precisely synchronise epithelial, endothelial, stromal, and immunological processes upon the embryo’s arrival in various contexts. The implantation window is established, and the endometrium is not saturated with supraphysiological hormones.

The behaviour of subgroups in RIF/FET studies further corroborates a story of precision utilisation. Early age may facilitate increased Treg expansion and more versatile receptor/epigenetic configurations; however, blastocyst-stage transfer generally magnifies advantages likely due to the synergistic effects of embryo-derived signals (including endogenous hCG) with the exogenous stimulus at the interface [13]. The notion of roughly 500 IU, characterised by high concentration, is implemented into dependable receptor engagement by methods that include fundal/mid-cavity catheter implantation, progressive instillation, and a short stay time before embryo loading. These procedures also improve boundary layer exposure and reduce reflux [7,120]. The correlation between window capture and improved outcomes is enhanced when clinics additionally focus on precise pharmacodynamics, such as monitoring alterations in VE-cadherin/CD146, ACTA2/NOTCH1/C3, or FOXP3^+^ Tregs/uNK. This reinforces the idea that hCG’s effectiveness in RIF/FET is contingent upon specific contexts rather than generally applicable [10,12,120].

The impact magnitude seems to vary according to the embryonic stage, which aligns with the underlying biological principles. In freeze-all protocols across all age demographics, D5 blastocysts demonstrate superior implantation and clinical pregnancy rates compared to D3 and D6 transfers [35]. D5 blastocysts demonstrate an increased likelihood of zona hatching, active paracrine secretion (including endogenous hCG), and a better-developed trophectoderm upon arrival. These elements augment the interaction with a pre-primed endometrium during the critical peri-transfer minutes [131]. The timing synergy allows a small-volume, high-concentration exogenous intrauterine hCG pulse to collaborate with the embryo’s signals, optimising microvascular shear as junctions tighten (VE-cadherin/CD146), accelerating early stromal decidual cues (ACTA2/NOTCH1/C3) at critical moments, and enhancing epithelial adhesion remodelling (rapid PI3K/Akt→eNOS and ADAM17→EREG/HB-EGF relays) [10]. D6 blastocysts may undergo temporal slippage, since the endometrium could be somewhat past its ideal receptivity, reducing additive effects. Conversely, D3 cleavage-stage embryos provide diminished and less specific endometrial training, leading to a smaller number of sites for the same exogenous pulse to “lock onto” [132]. Blastocyst transfers demonstrate the most pronounced clinical outcomes in recurrent implantation failure cohorts, especially in younger patients who possess more sensitive receptor and epigenetic profiles, as well as enhanced immunological plasticity, such as Treg expansion [69]. D5 embryos should be prioritised in future experiments. Administer about 500 IU in ≤500 μL at ≥2 IU/μL minutes before ET and conduct pharmacodynamic assessments to ensure that the hCG pulse and embryo-derived signals appropriately activate the relevant axis. The converging lines emphasise the need for synchronisation of stage timing [133].

They facilitate understanding of expectations and the timing for using hCG with other intrauterine therapies. A 2025 network meta-analysis indicated that PRP and PBMCs were superior compared to hCG in terms of clinical pregnancy and live birth rates. Nonetheless, G-CSF and hCG outperformed the placebo/control group. We interpret this order as phenotype and target selection rather than a dismissal of hCG [134]. The infusion of PBMCs directly modulates the immunological environment, which is potentially beneficial in scenarios where tolerance presents a significant obstacle, while PRP serves as a comprehensive trophic and angiogenic agent suitable for addressing substrate deficiencies, such as a thin, hypotrophic endometrium [135]. The time-lockable receptor signal hCG synchronises the epithelial, endothelial, stromal, and immunological axis just before transfer. The operational methodology is as follows. If the analysis indicates issues with cellularity or perfusion, provide PRP or G-CSF upstream to restore the substrate, followed by the application of hCG upon transfer to facilitate adhesion, vascular stability, and immunological tolerance [136]. If tolerance (characterised by low FOXP3^+^ Tregs and maladaptive uNK topology) or junctional cohesion (indicated by weak VE-cadherin/CD146) is the constraining factor, utilise hCG as the primary anchor and incorporate additional tools solely when on-target pharmacodynamic outcomes remain suboptimal (for instance, if the enhancement of VE-cadherin/CD146 is insufficient or Treg/uNK rebalancing is incomplete). This staged, deficit-oriented strategy uses the peri-transfer hCG pulse as the synchronising mechanism that transforms a competent substrate into a coordinated receptive state, minimises redundancy, and optimises the strengths of each modality [69,134].

Neutral or unfavourable trials reveal design factors that should influence future research. The lack of benefit from administering 48–72 or 24 h before ET dosing underscores the imperative for rigorous protocol standardisation regarding four pharmacologic parameters: dose (~500 IU), concentration (≥2 IU/µL), volume (≤500 µL), and pre-ET interval (measured in minutes, not days) [118,119]. It further allows the incorporation of on-target PD with live birth, guaranteeing that biological factors are clearly included during effectiveness assessment. PD panels must obtain samples from all four compartments: endothelial (VE-cadherin/CD146 junctional indices through digital histomorphometry), stromal (ACTA2/NOTCH1/C3 alongside Beclin1/LC3/p62 proteostasis equilibrium), epithelial (e.g., p-Akt/eNOS and miR-126-3p→PIK3R2 profiles), and immune (FOXP3+ Treg frequency/function, uNK density/topology). Minimally invasive peritoneal dialysis may obtain epithelial and soluble vascular/immune signals from uterine fluid 15–60 min after instillation [12,20,120].

Due to the influence of these factors on size and the potential for confounding, the trial design must pre-specify stratification based on age bands, cycle type (fresh versus programmed frozen embryo transfer), embryo stage (day 5 versus day 3), and baseline endometrial phenotype (LHCGR competence, autophagy flux, junctional integrity, immune set-point) [7,69,117]. Incorporating phenotypes defined by stromal stress, junctional fragility, uNK/Treg imbalance, or LHCGR deficiency would improve statistical efficiency and increase the prior for benefit in a biomarker-enriched randomisation. The exposure–response surface may be defined within arms by the micro-randomisation of timing (e.g., 5, 30, or 60 min pre-ET) and dosage variation (250, 500, or 1000 IU), while preserving a uniform concentration/volume framework. To guarantee exposure integrity, procedures must standardise the elimination of air bubbles, the infusion rate, the catheter location (fundal mid-cavity), and a short rest period prior to embryo loading. Per-protocol sensitivity analysis may be conducted by monitoring reflux and procedure timestamps [137].

The short, actionable duration of hCG necessitates objective verification of intervention fidelity. To define “on-target” exposure, we suggest using PK measures in uterine fluid (e.g., C_max, “dwell AUC”) with known PD criteria (e.g., ≥X% enhancement in VE-cadherin continuity; activation of ACTA2/NOTCH1/C3; ΔFOXP3^+^ Tregs). Employing epigenetic/EV readouts (H3K27Ac alterations at FOXO1/HOXA10/HAND2. EV proteins/miRNAs) and the epithelial PGF_2_α→ADAM17→EREG/HB-EGF pathway as investigative mediators facilitates causal mediation analyses that connect hCG to subsequent biological and clinical outcomes, surpassing simple correlation [60,123].

The main endpoint should be live birth, with core efficacy assessed by intention-to-treat analysis and hierarchical testing to manage multiplicity, whereas clinical pregnancy and implantation are regarded as important secondary outcomes. Instrumental–variable or two-stage models evaluate the complier average causal impact among PD-positive individuals, whereas co-primary PD (e.g., VE-cadherin/CD146 increase) may be analysed as the main surrogates. To mitigate noise, it is imperative to establish central labs for PD tests, conduct blinded imaging analyses, and implement pre-registered analytical strategies, including temporal bins, embryo-quality variables, PGT-A status, and consistency in luteal support. Safety monitoring must include cramping/bleeding, infection, and a few adverse events with established stopping criteria, despite prior evidence being encouraging [7,82,117].

Ultimately, there is no consistent rise in miscarriage, ectopic pregnancy, or multiple gestations in controlled comparisons, indicating that safety and practicality remain advantageous under standardised preparation and atraumatic approaches [7,82,117]. Mechanistically, hCG’s NO-permissive microflow and junctional maturation, absent VEGFR1/2 overexpression, limit oedema or haemorrhage [10,120]. These biology-aligned safety signals, context-specific effectiveness, and novel biomarker frameworks support the notion that intrauterine hCG is a precise, mechanism-based instrument. This involves careful implementation before transfer in programmed FET, focusing on blastocysts and selecting phenotypes with identifiable deficiencies in LHCGR signalling, endothelial cohesion, stromal proteostasis, or immune tolerance, followed by the confirmation of on-target engagement as part of standard quality control.

The findings of our investigation endorse a cohesive paradigm whereby intrauterine hCG functions as a temporal organiser of endometrial receptivity rather than serving as a singular route effector. hCG coordinates several cellular compartments throughout the limited implantation period by synchronising the regulation of immunological tolerance mechanisms, endothelial junctional integrity, stromal decidual viability, proteostasis, and epithelial adhesion capability. Significantly, these effects are synergistic rather than additive, suggesting that the disruption or misalignment of a single layer may reduce the total implantation response. Positioning hCG activity within this integrated network provides a conceptual framework that harmonises clinical variability with mechanistic variation. This provides a rational foundation for comprehending when, how, and in whom the addition of hCG to the uterus may be most beneficial.

## 8. Future Directions

Future avenues for intrauterine hCG research may be classified into initial mechanistic studies and immediate, therapeutically relevant approaches. This difference is crucial for the continuous improvement of the molecular basis of endometrial receptivity and its later use in clinical practice. The resulting image is one of intense, compartmentalised signalling superimposed atop epigenetic and paracrine reprogramming. This naturally results in a future research phase that is phenotype-selected and mechanism-based.

### 8.1. Clinically Actionable Directions

A primary objective is to formalise biomarker-enriched, PD-verified randomised trials in planned FET, ensuring regulated timing and endocrine tone. Compact phenotyping panels ought to be utilised for pre-stratifying enrolment. For vascular integrity, these are VE-cadherin/CD146; for stromal viability and proteostasis, these are ACTA2/NOTCH1/C3 and Beclin1/LC3/p62; for immune tolerance, these are FOXP3+ Tregs and uNK topology; and for epithelial–stromal readiness, these are HOXA10/ITGB3/LIF and FOXO1 [10,12,69,73]. Experimental comparison of peri-transfer dosing windows within each stratum (e.g., 5, 30, or 60 min before transfer) should be feasible, with a standardised backbone of approximately 500 IU, ≤500 μL, and ≥2 IU/μL. Co-primary pharmacodynamic objectives, such as endothelial junctional continuity, stromal survival/decidual induction, immune rebalancing, and epithelial p-Akt/eNOS or miR-126-3p/PIK3R2 signatures, should be integrated with live birth outcomes [10,20,69].

### 8.2. Research-Stage Exploratory Directions

Concurrent efforts should enhance the utility of scaled mechanistic readouts. Multicentre pharmacokinetic–pharmacodynamic maps of intrauterine exposure and duration may be produced using uterine fluid collection performed 15–60 min after hCG treatment [138]. This approach provides a minimally invasive framework for temporary epithelial, vascular, and immunological mediators. Pre- and post-instillation biopsies using single-cell and spatial multi-omics may clarify neighbourhood-level influences on uNK and endothelial cells, signalling biases (cAMP/PKA vs. ERK versus PI3K/Akt→eNOS), and cell-type-specific LHCGR participation [3,10,120]. To evaluate the potential of novel epigenetic and extracellular vesicle layers, specifically, EV cargo remodelling in decidualised stroma and H3K27Ac modifications at FOXO1/HOXA10/HAND2 as surrogate markers in adaptive designs, and to assess their statistical ability to communicate treatment effects on implantation endpoints, it is essential to advance from mere association to causal mediation [60].

In dosage formulation science, targeted research is advantageous. The impact of concentration, dwell duration, or carrier composition on biased signalling to improve endothelial junction stabilisation and epithelial nitric oxide generation, without receptor desensitisation, remains uncertain. Despite approximately 500 IU frequently being recognised as a viable threshold for multi-compartment engagement [10,120], small-volume, high-concentration boluses seem to optimise boundary–layer interaction. Systematic evaluations of protein carriers, pH/osmolality, and viscosity may optimise mucosal retention while avoiding contractions. Standardisation and auditing should be implemented for the following catheter techniques: gradual instillation, elimination of air bubbles, catheter placement in the fundal mid-cavity using ultrasound guidance, and a brief dwell period [139]. Reflux must be time-stamped to facilitate subsequent analysis according to the methodology.

Moreover, rather than using empirical additions, the next experiments should meticulously devise combination strategies. In the preparation for phenotypes marked by substrate deficiencies (thin, hypotrophic endometrium), upstream agents, such as PRP or G-CSF, may be sequenced. Peri-transfer hCG may be delivered thereafter to synchronise the epithelial–vascular–immune axis at the designated window [134]. Hyaluronan-enriched fluids applied with hCG priming may synchronise biochemical preparedness with near-wall fluid dynamics during apposition, even when adhesion mechanics are constrained despite continuous signalling [2]. Conversely, hCG should serve as the cornerstone in immune-dominant phenotypes marked by diminished Tregs or maladaptive uNK architecture. To prevent counter-phasic effects, further immune conditioning must depend on on-target immunological PD [13,73].

Model systems that preserve the context of human tissue will accelerate translation. The integration of an exogenous hCG pulse with embryo-derived signals and the capacity of D5 embryos to augment or prolong endometrial PD can be assessed through co-culture platforms that merge human blastocysts or trophoblast organoids with hormone-programmed endometrial epithelium/stroma under controlled shear conditions [35,123]. Microfluidic “uterine-on-chip” devices can regulate dwell duration, shear, and concentration to delineate exposure–response surfaces that cannot be examined in vivo with high resolution. To create a standardised currency between laboratory and clinical environments, these preclinical instruments must be actively linked to clinical indicators of Parkinson’s disease.

Ultimately, implementation research is essential to guarantee that accurate deployment enhances practical results. Despite the reassuring nature of the current data and the vascular biology’s opposition to oedema or haemorrhage in NO-stabilised junctions, it includes practical multicentre trials with centralised PD laboratories; cost-effectiveness modelling that assesses hCG relative to alternatives, like PRP and PBMC, within phenotype-defined populations; and safety registries that track cramping, bleeding, infection, and obstetric sequelae [7,10,82,117]. This research agenda could transform intrauterine hCG from a disparate adjunct into a validated, mechanism-based intervention, applicable when biological factors, timing, and embryonic development stage align, contingent upon standardised exposure, concurrent pharmacodynamics, and phenotype stratification.

## 9. Conclusions and Clinical Implications

Intrauterine hCG is an innovative precision instrument for implantation assistance that connects molecular processes to clinical use. The advantages are most evident in carefully chosen clinical settings, especially during frozen embryo transfer cycles, including blastocyst-stage embryos and customised endometrial preparation methods. Before embryo transfer, hCG seems to synchronise immunological, vascular, stromal, and epithelial readiness when delivered as a concentrated, short-interval infusion.

In the future, hCG should be used according to the patient’s phenotype. Patients exhibiting compromised immunological tolerance, inadequate vascular tone, or diminished stromal survival pathways should be prioritised above the use of hCG as a general adjuvant. Standardised techniques based on concentration, dwell duration, catheter technique, and pharmacodynamic evaluations will enable the shift of hCG from empirical use to focused intervention as mechanistic biomarkers develop.

In certain IVF cohorts, hCG may develop into a clinically relevant, mechanism-based treatment by carefully structured randomised trials, pharmacodynamic evaluation, and adherence to personalised embryo transfer protocols. The objective is to introduce a layer of molecular synchronisation grounded in evidence at the moment of implantation, rather than to supplant existing approaches. This will advance assisted reproduction towards a more physiologically precise epoch.

## Figures and Tables

**Table 1:** Mechanistic map of intrauterine hCG across endometrial compartments.

Compartment	LHCGR Presence and Proximal Signalling	Very-Early Effects (0–60 min)	Early Transcriptional/Epigenetic Programs (2–24 h)	Paracrine/Secretome and EV Outputs	Practical PD Readouts (Sample and Assay)	Clinical Implication (How This Helps Implantation)
Luminal epithelium [2,8,20,60]	LHCGR→Gs/cAMP/PKA; bias to PI3K/Akt→eNOSvia miR-126-3p→PIK3R2↓ (Wang). hCG also upregulates cPLA2/COX/AKR1C3 → PGF_2_α (Jin).	↑ p-Akt (Ser473), ↑ p-eNOS (Ser1177) → NO burst; membrane/cortical actin softening; rapid adhesion-competence(integrin activation/clustering). PGF_2_α surge → ADAM17 activation → EREG/HB-EGF shedding (Jin; Wang).	Induction of HOXA10/FOXO1 target programs; enhancer opening marks (H3K27Ac) at receptivity loci (Žukauskaitė); coordinated integrin β3 licensing and LIF axis tuning (Makrigiannakis).	EGFR ligands (EREG/HB-EGF), VEGF, chemokines (CCL4, IL-1β/IL-6/IL-17) in mixed cultures (Srivastava); EV miRNAs (miR-340-3p, miR-663a, miR-766-5p, etc.) and proteins that condition stroma/endothelium/immune (Žukauskaitė).	Uterine fluid at 15–60 min: NOx, EREG/HB-EGF, PGF_2_α (ELISA/LC-MS); epithelial brush/aspirate: p-Akt/p-eNOS (WB/IF), miR-126-3p/PIK3R2 (qPCR).	Faster apposition→adhesiontransition; improved microlubrication and shear tuning at the interface; paracrine priming of stromal decidual response.
Endothelium/microvasculature [2,8,10]	LHCGR on endometrial endothelium (Makrigiannakis); coupling to PI3K/Akt–eNOS; junctional signalling nodes (VE-cadherin/β-catenin/p120).	Selective junctional maturation: ↑ VE-cadherin (CD144) and CD146 (MCAM) without VEGFR1/2 change → tighter adherens contacts; reduced permeability without sprouting (Bienert). NO/sGC/cGMP smooths subendothelial flow and stabilises junctions.	Transcriptional reinforcement of junctional complexes; restraint of Src-phosphorylation at VE-cadherin; endothelial quiescence signature compatible with controlled permeability.	Angiocrine tuning (VEGF modest), chemokines guiding trophoblast navigation; low-noise vascular milieu that supports cytokine/nutrient exchange.	Biopsy IF with digital morphometry: VE-cadherin continuity index, CD146 density; uterine fluid: vWF fragments low, stable VEGF; Doppler microflow indices (optional).	Shear-stable, non-edematous bed for apposition; constrains trophoblast entry to controlled, non-destructive invasion.
Stroma/decidua (ESCs) [8,12,60]	LHCGR→cAMP/PKA, ERK1/2, mTOR, autophagy nodes (Beclin1/LC3/p62) (Wang 2024 [8]). Crosstalk with NOTCH and complement.	Early survival reset: induction of ACTA2 (α-SMA) in subepithelial/perivascular stromal cells; pro-survival tone; initiation of decidual trajectory (Strug).	↑ HOXA10, ITGB3, FOXO1, LIF, L-selectin ligands; ERK1/2 and mTOR activation; autophagy restoration (↑ Beclin1/LC3, ↓ p62). Epigenetic priming: ↑ H3K27Ac at FOXO1/HOXA10/HAND2; shifts in TET/HDAC/EED (Žukauskaitė).	EV cargo remodelling (IGFBP1/7, PAPP-A, MMP-2, STC-1/2; miRNAs) that communicates receptivity and immune tone (Žukauskaitė).	Biopsy: ACTA2/NOTCH1/C3 IF; WB/qPCR for HOXA10/FOXO1/ITGB3/LIF; autophagy markers (LC3-II/p62); ChIP-qPCR for H3K27Ac (exploratory); EV profiling (MS/miRNA-seq) from uterine fluid.	Raises decidual robustness and proteostasis; improves resistance to oxidative/metabolic stress during early invasion; aligns stromal timing with epithelial/vascular signals.
Innate and adaptive immune (uNK, Tregs, DC/MΦ) [1,3,20,33]	LHCGR on immune subsets variably reported; robust indirect modulation via epithelial/stromal signals and endocrine exposure.	Bias toward tolerance: rapid cytokine milieu shift favouring IL-10/TGF-β; uNK functional tilt to pro-angiogenic, matrix-modulating phenotype; APC checkpoint up-tuning (PD-L1/IDO1) (Schumacher; Lee; Zhou).	Expansion of FOXP3^+^ Tregs; stable uNK topology supportive of spiral artery remodelling; reduced Th1/Th17 noise; coordinated chemokine gradients for trophoblast guidance.	Secretome changes from epithelium/stroma (CCL4, IL-1β/6/17, VEGF, HGF, FGF2) orchestrate immune positioning (Srivastava); EV-mediated immune instruction (Žukauskaitė).	Peripheral blood: %FOXP3^+^ Tregs (flow) pre-/post-instillation; endometrial aspirate: uNK (CD56^bright^CD16^−^) density/topology (IF/CyTOF); DC/MΦ PD-L1/IDO1 (IHC/flow).	Reduces rejection-like noise; facilitates vascular remodelling and controlled trophoblast migration; correlates with improved implantation/LBR in RIF/FET cohorts (Liu).
Cross-compartment relays [8,10,12,60,61]	hCG→epithelial PGF_2_α → ADAM17→EREG/HB-EGF → stromal EGFR; epithelium miR-126-3p/PIK3R2 gating PI3K/Akt→eNOS→vascular NO.	Temporal stacking of fast (sec–min) NO/junctional edits with intermediate (10–60 min) EGFR ligands and early (2–6 h) stromal survival/decidual waves.	Chromatin priming (H3K27Ac) sustains a permissive state into early invasion; EV cargo propagates instructions across compartments (Žukauskaitė).	Integrated secretome/EV dialogue maintains window-specific synchrony.	Composite PD panel across matrices as above; time-stamped sampling (15–60 min; 2–6 h; 24 h).	Explains why minutes before ET bolus (≤500 μL; ≥2 IU/μL; ~500 IU) performs better than day-before dosing; supports precision timing in FET/RIF.

Table 1 delineates the compartment-resolved mechanistic map of intrauterine hCG within the peri-implantation endometrium, including paracrine/EV outputs, initial transcriptional/epigenetic programs (2–24 h), proximal LHCGR signalling, immediate (0–60 min) effects, potential pharmacodynamic readouts, and clinical ramifications. Abbreviations: LHCGR, luteinising hormone/hCG receptor; EV, extracellular vesicle; uNK, uterine natural killer cell; DC, dendritic cell; MΦ, macrophage; IF, immunofluorescence; IHC, immunohistochemistry; WB, Western blot; ChIP, chromatin immunoprecipitation; NO, nitric oxide; EGFR, epidermal growth factor receptor; ACTA2, alpha-smooth muscle actin; ADAM17, a disintegrin and metalloproteinase domain-containing protein 17; Akt, protein kinase B; cAMP, cyclic adenosine monophosphate; COX, cyclooxygenase; eNOS, endothelial nitric oxide synthase; ERK, extracellular signal-regulated kinase; ESCs, endometrial stromal cells; FOXO1, forkhead box O1; HB-EGF, heparin-binding epidermal growth factor-like growth factor; IL, interleukin; ITGB3, integrin beta-3; LC3, microtubule-associated protein 1 light chain 3; LIF, leukemia inhibitory factor; MCAM, melanoma cell adhesion molecule (CD146); miRNA, microRNA; mTOR, mechanistic target of rapamycin; PD, pharmacodynamic; PGF_2_α, prostaglandin F2 alpha; PI3K, phosphoinositide 3-kinase; PIK3R2, phosphoinositide-3-kinase regulatory subunit 2; PKA, protein kinase A; qPCR, quantitative polymerase chain reaction; RIF, recurrent implantation failure; Treg, regulatory T cell; VEGF, vascular endothelial growth factor.

**Table 2:** Preclinical and translational evidence base underpinning intrauterine hCG.

Study (Year)	Model/System	Exposure (Dose, Timing, Format)	Principal Readouts	Direction of Effect	Mechanistic Pathway Implicated	Key Limitations	Translational Takeaway
Wang (2024) [8]	EID mouse model (mifepristone) + human endometrial epithelial cells (EECs)	hCG treatment in vivo and in vitro (as per study protocols)	↑ miR-126-3p; ↓ PIK3R2; ↑ p-PI3K (p85α), p110α, p-Akt, p-eNOS; ↑ EEC proliferation; miR-126-3p inhibition blunted effects	Pro-receptivity; epithelial proliferation and signalling activation	miR-126-3p → PIK3R2 ↓ → PI3K/Akt → eNOS	Rodent model; epithelial focus; dose–time external validity	Validates a causal epithelial axis that can be assayed rapidly after peri-transfer hCG
Bienert (2021) [10]	Human endometrium at WOI after intrauterine hCG flush vs. no hCG	Single intrauterine instillation during WOI	↑ VE-cadherin (CD144), ↑ CD146 (MCAM); no change in VEGFR1/2, CD31; epithelial, stromal, immune markers largely unchanged	Endothelial junctional stabilisation without neovascularisation	Adherens junction maturation; NO-permissive flow synergy plausible	Small n; flow cytometry phenotyping; no clinical endpoints	Supports vascular “tightening” as the dominant microvascular effect of hCG
Srivastava (2022) [62]	3D primary human endometrial epithelial, stromal, and mixed cultures	rhCG 0–100 IU/mL for 24 h	Epithelial: HGF/M-CSF (macropinocytosis); stromal: CCL4, FGF2, IL-1β/6/17, VEGF; mixed: EMT-like (FGF2, HGF, IL-1β, TNF)	Context-specific secretome reprogramming	Paracrine conductorrole of hCG across compartments	In vitro duration; wide cytokine panel (multiplicity)	Provides the cytokine blueprint for hCG’s compartment-specific paracrine tuning
Strug (2016) [12]	RCT in oocyte donors; endometrial biopsies post-instillation	500 IU intrauterine, day 3 equivalent; biopsy ~48 h later	↑ ESR1/PGR; ↑ ACTA2 (α-SMA), C3, NOTCH1; ERA not globally shifted; gland–stroma resynchronisation	Early decidual/survival programming; stromal timing reset	NOTCH/complementengagement; stromal survival tone	Small donor cohort; single bolus; not powered for clinical endpoints	Mechanistic RCT evidence that peri-transfer hCG re-phases stroma toward receptivity
Žukauskaitė (2025) [60]	Human ESCs (decidualised) ± hCG; multi-omics	hCG 24 h ± decidualisation (time-staged)	↑ PRL; ↓ IL6/BAK1; ↑ H3K27Ac at FOXO1/HOXA10/HAND2; shifts in EED/HDAC1/TET1-3; EV proteins (IGFBP1/7, PAPP-A, MMP-2, STC-1/2) and miRNAs remodeled	Epigenetic priming + EV paracrine export	Chromatin opening at decidual enhancers; EV-mediated signalling	Small sample sizes; in vitro ESCs	hCG imprints a durable, transmissible receptivity signal (chromatin + EVs)
Jin (2008) [61]	Human endometrial epithelium → stromal decidualisation relay	hCG to epithelium; stromal readouts	↑ cPLA2/COX-1/COX-2/AKR1C3 → PGF_2_α; ADAM17-dependent shedding of EREG/HB-EGF; ↑ IGFBP1 during decidualisation	Upstream epithelial prostaglandin–EGFR relay	PGF_2_α → ADAM17 → EREG/HB-EGF → EGFR → decidualisation	In vitro; needs in vivo correlation	Explains why minutes before ETdosing can prime stroma via epithelial relay
Giuliani (2015) [14]	Fertile donors; endometrium after single intrauterine hCG	Single IU hCG during ET-equivalent window	Histologic redistribution of uNK subsets (CD56/CD16)	Immune topology shift toward an implantation-supportive state	uNK re-patterning via local cues	Histologic endpoints; fertile donors	Shows that hCG can quickly reshape the immune landscape at the interface
Wang (2024) [8]	Human ESCs from controls vs. RIFpatients ± hCG	ESCs treated with hCG; LHCGR knockdown rescue	↑ LHCGR signalling; ↑ HOXA10, ITGB3, FOXO1, LIF, L-selectin ligands; ↑ Beclin1/LC3, ↓ p62; ERK1/2 and mTOR activation; LHCGR siRNA blunts effects	Restores receptivity and autophagy; modulates apoptosis	LHCGR→ERK/mTOR + autophagy (Beclin1/LC3/p62)	Small n; in vitro; apoptosis markers complex	Identifies RIF-typical deficits and shows they are hCG-responsive ex vivo

Table 2 presents the models, exposure circumstances, primary outcomes, impact direction, molecular pathways, limitations, and translational implications of preclinical and translational research linking intrauterine/embryo-mimetic hCG to endometrial signalling, structure, and immunological profile. Abbreviations: ACTA2, alpha-smooth muscle actin; Akt, protein kinase B; CD, cluster of differentiation; EGFR, epidermal growth factor receptor; eNOS, endothelial nitric oxide synthase; ERK, extracellular signal-regulated kinase; ESCs, endometrial stromal cells; EV, extracellular vesicle; FOXO1, forkhead box O1; H3K27Ac, histone H3 lysine 27 acetylation; HB-EGF, heparin-binding epidermal growth factor-like growth factor; IL, interleukin; ITGB3, integrin beta-3; LC3, microtubule-associated protein 1 light chain 3; LHCGR, luteinising hormone/choriogonadotropin receptor; LIF, leukemia inhibitory factor; mTOR, mechanistic target of rapamycin; p-Akt, phosphorylated Akt; p-eNOS, phosphorylated endothelial nitric oxide synthase; PI3K, phosphoinositide 3-kinase; PIK3R2, phosphoinositide-3-kinase regulatory subunit 2; RCT, randomied controlled trial; RIF, recurrent implantation failure; uNK, uterine natural killer cell; VEGF, vascular endothelial growth factor.

**Table 3:** Clinical evidence landscape for intrauterine hCG.

Study	Design and N	Population/Context	Timing vs. ET	Dose (IU)	Volume/Conc.	Embryo Stage	Primary Outcomes Reported	Net Effect on Key Endpoints	Safety Notes	Notes
Gao (2023) [82]	Meta-analysis of 15 RCTs, N = 2763	Mixed IVF populations (fresh + FET)	Mixed across trials; subgroup highlights ≤ 15 min	Varied; subgroup signal at ~500	Varied; subgroup: ≤500 μL, ≥2 IU/μL	Mixed (D3/D5)	↑ LBR, ↑ OPR, ↑ CPR, ↑ IR; ↓ MR	Positive overall; strongest when ~500 IU given close to ET	No consistent increase in adverse events	Identifies dose–timing sweet spot (500 IU within 15 min)
Hou (2018) [117]	Meta-analysis of 6 RCTs (fresh only), N = 2759	Fresh ETcycles	Mostly day of ET; variable	Varied	NR	Mixed (often blastocyst)	↑ Biochemical, ↑ ongoing; no sig. change in CPR, IR, LBR	Neutral for clinical endpoints in fresh	No consistent harms	Suggests fresh context blunts translation to LBR/CPR
Luo (2024) [7]	Meta-analysis, 13 studies, N = 2157	RIF (per ESHRE 2023), mostly FET	Mixed; subgroup best when immediately pre-ET	~500 favoured	≤500 μL, ≥2 IU/μL favoured	D3/D5; many blastocysts	↑ CPR, ↑ IR; LBR modest ↑ but underpowered	Positive in RIF; best with micro-volume, higher conc., ~500 IU	No ↑ in miscarriage, ectopic, multiples	Context-sensitive efficacy in RIF/FET
Mustapha (2022) [118]	Randomised prospective (three-arm), N = 174	Fresh, autologous blastocyst ET	−24 h (day before)	500 or 1000 vs. control	NR	D5	CPR, IR	Neutral: no improvement vs. control	NR	Long lead-time likely misses the actionable window
Gedela (2022) [119]	RCT (freeze-all program), N = 180 (100 vs. 80)	FET (two good-grade blastocysts transferred)	−48 to −72 h	500 vs. no hCG flush	NR	D5	CPR, IR, MPR	Neutral (CPR 71% vs. 76%, *p* = 0.45)	Miscarriage/LBR NR	Earlier dosing (2–3 days) is not helpful
Miao Wang (2019) [120]	RCT, N = 140	RIF, FET	~3 min pre-ET	hCG in 40 μL	40 μL (high conc.)	Mixed (likely D5 emphasis)	↑ IR, ↑ CPR	Positivewith micro-volume, immediate timing	No safety signal	Supports small volume/immediateinstillation
Liu (2019) [13]	Prospective cohort, N ≈ 305 (152 vs. 153)	RIF, FET	−3 days before ET	500	Diluted in saline	Blastocyst subgroup notable	↑ IR, ↑ CPR, ↑ LBR; ↑ peripheral Tregs	Positive, esp. in younger and blastocysttransfers	NR	Immune PD (Treg ↑) as a correlate of benefit
Strug (2016) [12]	RCT (mechanistic), oocyte donors, n = 15	Donor cycles; biopsy endpoints	Day 3 equivalent; biopsy ~48 h later	500	NR	NA (biopsy study)	↑ ESR1/PGR; ↑ ACTA2/C3/NOTCH1; synchrony	Mechanistic positive; no clinical endpoints	No harms	Shows stromal survival/decidual induction
Bienert (2021) [10]	Prospective tissue study, n = 12	Natural/primed WOI; hCG flush vs. none	Single intrauterine at WOI	NR	NR	NA (tissue phenotyping)	↑ VE-cadherin (CD144), ↑ CD146; VEGFR1/2 unchanged	Mechanistic-positive (junctional maturation)	NA	Vascular tightening, not sprouting

Controlled clinical trials and meta-analyses of intrauterine hCG administration prior to embryo transfer classified by exposure parameters, embryo developmental stage, safety, results, timing in relation to embryo transfer, and cycle context. NR signifies “not reported.” Abbreviations: CPR, clinical pregnancy rate; D3, day 3 embryo; D5, day 5 blastocyst; ET, embryo transfer; ESHRE, European Society of Human Reproduction and Embryology; FET, frozen embryo transfer; hCG, human chorionic gonadotropin; IR, implantation rate; IU, international units; LBR, live birth rate; MPR, multiple pregnancy rate; MR, miscarriage rate; NR, not reported; OPR, ongoing pregnancy rate; RCT, randomised controlled trial; RIF, recurrent implantation failure; Treg, regulatory T cell; WOI, window of implantation.

**Table 4:** Practical “exposure recipe” for intrauterine hCG: standardised parameters, mechanistic rationale, supporting evidence, quality assurance notes, and on-target PD checks.

Parameter	Recommended Setting	Mechanistic Rationale	Practical QA/Technique	Suggested PD Check (Matrix and Assay)
Dose [7,69,82]	~500 IU	Threshold for multi-compartment LHCGR engagement (epithelium, stroma, endothelium; uNK/APC indirectly) without desensitisation; repeatedly emerges in positive subgroups.	Use unit-verified vial; avoid multi-use vials; document lot.	Uterine fluid at 15–60 min: detect EREG/HB-EGF/NOx; epithelial brush: p-Akt/p-eNOS (WB/IF).
Concentration [82,124]	≥2 IU/μL	Higher ligand activity at the mucosal boundary layer improves receptor occupancy and reduces immediate dilution.	Prepare in minimal carrier volume; verify final μL and IU.	Short-interval rise in epithelial p-Akt or miR-126-3p ↑/PIK3R2 ↓ (qPCR).
Volume [7,120]	≤500 μL (micro-bolus)	Small volume limits reflux, prolongs mucosal dwell, and preserves high local concentration; favours fast epithelial/vascular windows.	Prime catheter; slow instillation over ~10–20 s; avoid air bubbles; observe for reflux.	If available, note dwell time; optional ultrasound tip visualisation.
Timing vs. ET [10,118,119,120,123]	Minutes before ET (5–30 min ideal; ≤60 min)	Captures very early events (NO burst, VE-cadherin/CD146 tightening, ADAM17→EREG/HB-EGF relay) that decay within hours.	Sequence: instill hCG → wait brief dwell → load catheter → ET; timestamp both.	Uterine fluid 15–60 min post-instillation for PGF_2_α/EREG/HB-EGF; endothelial IF (biopsy only if research).
Cycle context [117]	Programmed FET (avoid fresh for this intervention)	Stable endocrine tone, predictable WOI; avoids supraphysiologic steroids and endometrial advancement that desynchronise PD.	Standardise E2/P dosing and luteal support; record P4 at ET.	Clinical PD: IR/CPR uplift more likely; optional Treg % change in blood (flow).
Embryo stage [35,69]	D5 blastocyst	Stronger embryo-derived cues (incl. endogenous hCG), zona hatching, better synergy with peri-transfer pulse.	Align lab scheduling to enable hCG→ET within 5–30 min for D5.	Higher amplitude PD expected across axes; monitor IR/CPR per stage.
Catheter position [120]	Fundal/mid-cavity, avoid touching the endometrium	Maximises spread, minimises focal trauma and contractions; reduces reflux down the cervix.	Ultrasound-guided soft catheter; no tenaculum if possible.	Procedural QA: note position, ease, and any bleeding/cramping.
Safety screen [82,117,124]	Rule out infection; avoid active bleeding/irritability	Reduces risk of ascending infection or cramping; maintains clean PD.	Quick checklist: afebrile; clean speculum exam; no purulent discharge.	Record AEs within 24–48 h (cramps/bleeding/fever).
Mechanistic PD panel [8,10,12,60,69,73,123]	Compact, matrix-matched set	Confirms on-target engagement for QC and research.	See right →.	Epithelium: p-Akt/p-eNOS, miR-126-3p/PIK3R2. Endothelium: VE-cadherin/CD146 continuity (IF). Stroma: ACTA2/NOTCH1/C3; LC3/p62. Immune: FOXP3^+^ Tregs (blood), uNK topology (aspirate IF).

A comprehensive “exposure recipe” for intrauterine hCG, including standardised parameters, supporting data, quality assurance comments, mechanistic rationale, and PD assessments that are accurate. Abbreviations: AEs, adverse events; APC, antigen-presenting cell; CPR, clinical pregnancy rate; D5, day 5 blastocyst; E2, estradiol; EREG, epiregulin; ET, embryo transfer; FET, frozen embryo transfer; hCG, human chorionic gonadotropin; HB-EGF, heparin-binding epidermal growth factor-like growth factor; IF, immunofluorescence; IR, implantation rate; IU, international units; LC3, microtubule-associated protein 1 light chain 3; LHCGR, luteinising hormone/choriogonadotropin receptor; miR, microRNA; NOx, nitric oxide metabolites (nitrite/nitrate); p-Akt, phosphorylated Akt; p-eNOS, phosphorylated endothelial nitric oxide synthase; P4, progesterone; PD, pharmacodynamic; PGF_2_α, prostaglandin F2 alpha; PIK3R2, phosphoinositide-3-kinase regulatory subunit 2; qPCR, quantitative polymerase chain reaction; QA, quality assurance; Treg, regulatory T cell; uNK, uterine natural killer cell; WB, Western blot; WOI, window of implantation; μL, microlitre.

## Data Availability

No new data were created or analyzed in this study. Data sharing is not applicable to this article.

## Abbreviations

ACTA2, alpha-smooth muscle actin; ADAM17, a disintegrin and metalloproteinase domain-containing protein 17; AEs, adverse events; Akt, protein kinase B; APC, antigen-presenting cell; cAMP, cyclic adenosine monophosphate; CD, cluster of differentiation; ChIP, chromatin immunoprecipitation; COX, cyclooxygenase; CPR, clinical pregnancy rate; DC, dendritic cell; D3, day 3 embryo; D5, day 5 blastocyst; E2, estradiol; EGFR, epidermal growth factor receptor; eNOS, endothelial nitric oxide synthase; EREG, epiregulin; ERK, extracellular signal-regulated kinase; ESCs, endometrial stromal cells; ESHRE, European Society of Human Reproduction and Embryology; ET, embryo transfer; EV, extracellular vesicle; FET, frozen embryo transfer; FOXO1, forkhead box O1; H3K27Ac, histone H3 lysine 27 acetylation; HB-EGF, heparin-binding epidermal growth factor-like growth factor; hCG, human chorionic gonadotropin; IF, immunofluorescence; IHC, immunohistochemistry; IL, interleukin; IR, implantation rate; ITGB3, integrin beta-3; IU, international units; LC3, microtubule-associated protein 1 light chain 3; LBR, live birth rate; LHCGR, luteinising hormone/choriogonadotropin receptor; LIF, leukemia inhibitory factor; MCAM, melanoma cell adhesion molecule; miR, microRNA; mTOR, mechanistic target of rapamycin; MPR, multiple pregnancy rate; MR, miscarriage rate; NO, nitric oxide; NOx, nitric oxide metabolites (nitrite/nitrate); NR, not reported; OPR, ongoing pregnancy rate; P4, progesterone; PD, pharmacodynamic; PGF_2_α, prostaglandin F2 alpha; PI3K, phosphoinositide 3-kinase; PIK3R2, phosphoinositide-3-kinase regulatory subunit 2; PKA, protein kinase A; qPCR, quantitative polymerase chain reaction; QA, quality assurance; RCT, randomised controlled trial; RIF, recurrent implantation failure; Treg, regulatory T cell; uNK, uterine natural killer cell; VEGF, vascular endothelial growth factor; WB, Western blot; WOI, window of implantation; μL, microlitre.
